# Geomorphically controlled coral distribution in degraded shallow reefs of the Western Caribbean

**DOI:** 10.7717/peerj.12590

**Published:** 2022-03-14

**Authors:** Alexis Enrique Medina-Valmaseda, Paul Blanchon, Lorenzo Alvarez-Filip, Esmeralda Pérez-Cervantes

**Affiliations:** 1 Posgrado en Ciencias del Mar y Limnología, Universidad Nacional Autónoma de México, Coyoacán, Ciudad de México, Mexico; 2 Reef Geoscience Group, Unidad Académica de Sistemas Arrecifales Instituto de Ciencias de Mar y Limnología, Universidad Nacional Autónoma de México, Puerto Morelos, Quintana Roo, Mexico; 3 Biodiversity and Reef Conservation Laboratory, Unidad Académica de Sistemas Arrecifales Instituto de Ciencias de Mar y Limnología, Universidad Nacional Autónoma de México, Puerto Morelos, Quintana Roo, Mexico

**Keywords:** Mesoamerican coral reef system, Geomorphic zonation, Coral species distribution patterns

## Abstract

The development of coral reefs results from the interaction between ecological and geological processes in space and time. Their difference in scale, however, makes it difficult to detect the impact of ecological changes on geological reef development. The decline of coral cover over the last 50 years, for example, has dramatically impaired the function of ecological processes on reefs. Yet given the limited-resolution of their Holocene record, it is uncertain how this will impact accretion and structural integrity over longer timescales. In addition, reports of this ecological decline have focused on intrinsic parameters such as coral cover and colony size at the expense of extrinsic ones such as geomorphic and environmental variables. Despite these problems, several attempts have been made to predict the long-term accretion status of reefs based entirely on the contemporary health status of benthic communities. Here we explore how this ecological decline is represented within the reef geomorphic structure, which represents the long-term expression of reef development. Using a detailed geomorphic zonation scheme, we analyze the distribution and biodiversity of reef-building corals in fringing-reef systems of the Mesoamerican Reef tract. We find a depth-related pattern in community structure which shows that the relative species distribution between geomorphic zones is statistically different. Despite these differences, contemporary coral assemblages in all zones are dominated by the same group of pioneer generalist species. These findings imply that first, coral species distribution is still controlled by extrinsic processes that generate the geomorphic zonation; second, that coral biodiversity still reflects species zonation patterns reported by early studies; and third that dominance of pioneer species implies that modern coral assemblages are in a prolonged post-disturbance adjustment stage. In conclusion, any accurate assessment of the future viability of reefs requires a consideration of the geomorphic context or risks miscalculating the impact of ecological changes on long-term reef development.

## Introduction

The declining cover of reef-building corals on modern reefs has become a global concern for both scientists and citizenry. The gravity of the situation became clear after only a decade of systematic monitoring, when a series of acute disease outbreaks decimated the dominant reef-building acroporid corals in the Caribbean (Knowlton et al., 1981; Gladfelter, 1982; Hughes, 1994; Aronson & Precht, 2001). Along with destruction caused by multiple strikes from intense hurricanes (*e.g*., Woodley et al., 1981), these outbreaks were quickly followed by regional mass mortality of algal-grazing Diademid urchins, which resulted in macroalgal blooms (Lessios et al., 1984; Gladfelter, 1982; Lewis, 1984). By the 1990s acroporid reefs began suffering climate-induced mass-bleaching episodes and disease epidemics also began affecting other major reef-builders (orbicellids) that had survived previous disturbances (Muller et al., 2010; Weil, 2004; Bruckner & Bruckner, 2006). These losses from disease and destruction were exacerbated by overfishing and nutrient pollution resulting from widespread and uncontrolled coastal development (Grigg & Dollar, 1990; Rogers & Beets, 2001; Hughes et al., 2003). By the turn of the century, surveys were reporting a regional decline in coral cover of more than 50% across the Western-Atlantic Reef Province (Gardner et al., 2003; Schutte, Selig & Bruno, 2010; Jackson et al., 2014; Cramer et al., 2020), producing a functional homogenization of coral species and a flattening of reef structure (*e.g*., Álvarez Filip et al., 2009; González-Barrios, Cabral-Tena & Alvarez-Filip, 2021). Understanding the extent of this decline on a regional scale, however, has relied exclusively on the data pooling from limited local observation, and this has led to significant uncertainty regarding its principal cause (Murdoch & Aronson, 1999; Wood, 2007; Jackson et al., 2014).

It is well understood that pooling ecological data from local studies can lead to inaccurate results, predict potential pseudo-trends, or misinterpret ecological processes because of scale changes in the analysis (Guzmán, Jackson & Weil, 1991; Karlson & Hurd, 1993; Williams et al., 2015b; Medina-Valmaseda et al., 2020; Dietzel et al., 2020). This is because regional meta-analyses extrapolate from small-scale coral abundance and coverage data gathered with different primary objectives in mind. A common problem, for example, is the relation between sampling scale and sensitivity of the observed ecological changes (*e.g*., Edmunds & Bruno, 1996), with extrapolation from small-to large-scale being performed without explicit sampling at new scales (Wiens, 1989). Furthermore, although regional meta-analyses account for inconsistencies among the various survey methods (Côté et al., 2005), they cannot account for inconsistencies resulting from omissions. The omission of the geomorphic context of ecological data by local studies, for example, can lead to inaccuracies in abundance-based data and thus misrepresent long-term spatial complexity and the ecological functioning of coral reefs at larger scales (*e.g*., Jackson et al., 2014; Williams et al., 2015b; Medina-Valmaseda et al., 2020).

Although elucidating the role of abiotic factors in coral community structure is challenging because of these scale differences (Murdoch & Aronson, 1999), separating ecological data from its geomorphic context risks obscuring spatial trends (*e.g*., Williams et al., 2015b). From an ecological perspective alone, the use of single ecological metrics, such as coral cover, to evaluate heterogeneous processes at regional scales gives an overly simplistic representation of reef structure (Viehman, Thur & Piniak, 2009; Hughes et al., 2010; Kuffner & Toth, 2016). A better understanding of the relationship between biotic data and its geomorphic context at larger scales, however, might be useful in interpreting the real signal of functional changes and improving long-term predictions of coral reefs and their response to global threats. Accretion potential, for example, has been reported to be heterogeneous among geomorphic zones (Perry, 1999; Blanchon et al., 2017) and also species-dependent (Perry & Alvarez-Filip, 2019; González-Barrios, Cabral-Tena & Alvarez-Filip, 2021). This issue is relevant at larger temporal scales because not all coral species contribute equally to reef accretion (cf. Macintyre & Glynn, 1976; Kuffner et al., 2019; Toth et al., 2019) and there are some biases and uncertainties in linking contemporary coral patterns with accretion potential (Wood, 2007).

Similarly, the use of the geomorphic zones to contextualize ecological indexes within restoration efforts might improve the outcome and enhance economic efficiency. Restoration efforts, for example, commonly lack geomorphological framework analysis in species distribution and omit the role of large-scale extrinsic factors, such as exposure to wave energy and reef degradation state. Such factors have been highlighted as the cause of restoration failures, in spite of the results being evaluated solely based on live coral coverage (Hein et al., 2020). To place this in context, it is estimated that the cost of Caribbean restoration programs varies between USD 10,000 ha^−1^ for the simplest transplant efforts and more than USD 2.0–6.5 million ha^−1^ in more complex projects involving the physical restoration of the reef framework (Spurgeon, 2001; Edwards, 2010) with a mean cost around USD 400,000 ha^−1^ (2010 USD; Bayraktarov et al., 2019). Given its variable history of success, the economic investment appears to be excessive in comparison to the modest results achieved in coral survival (∼60%), duration (1–2 years) and extent (0.01 ha or 108 m^2^; Bayraktarov et al., 2019).

Here we investigate whether geomorphically-controlled patterns in the decline of coral cover reported by Medina-Valmaseda et al. (2020) can be extended to the entire shallow geomorphic framework of the Mesoamerican reef tract. Using a standard geomorphic scheme and multi-year coral-cover and composition data, we analyze regional patterns in coral distribution and biodiversity. Then, using GIS-based environmental data, we explore how these patterns are influenced by key environmental controls on reef geomorphology: depth, wave exposure and hurricane incidence. We find that contemporary coral assemblages are dominated by the same group of pioneer species, but that their distribution between geomorphic depth zones is statistically different, and has not been completely homogenized by the decline in reef condition over the last 50 years.

## Methods

### Study region and geomorphic framework

**Figure 1:**
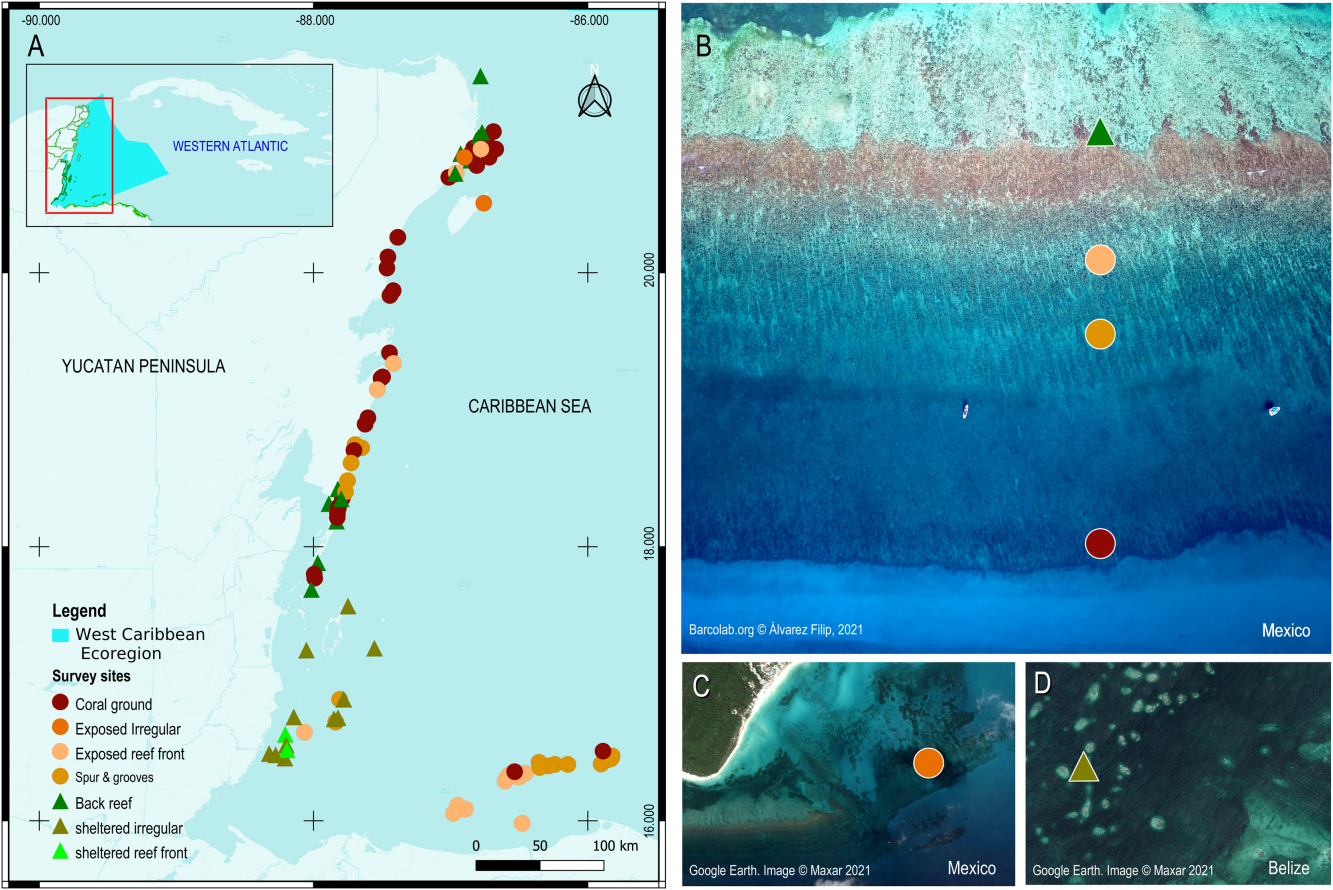
Survey sites and their geomorphic zones in the Mesoamerican reef tract (MART). (A) The MART is located in the West Caribbean ecoregion (represented by the irregular light blue polygon in the inset). (B–D) Reef sites are represented by colored symbols that correspond either to their benthic geomorphic zone and/or wave-energy designation. Map product was created with QGIS 3.14 Pi (QGIS Development Team, 2020).

The Mesoamerican Reef Tract (MART), located within the Western Caribbean marine ecoregion, stretches ∼1,000 km along the coasts of the Yucatan peninsula and northeast sector of Central America (Fig. 1; Spalding et al., 2007). It is composed of shallow detached fringing reefs that consist of two main geomorphic zones, a protected back-reef zone and an exposed reef-front zone separated by a crestline where waves break. These breakwater structures are limited to shallow water (<10 m deep) and are developed over and adjacent to a bedrock terrace which is veneered by coral grounds (Rodríguez-Martínez et al., 2011). Together the reef and terrace form a relatively consistent seascape over the inner fore-reef shelf (<15 m) throughout the MART (Blanchon et al., 2017). The shallow geomorphic framework of the MART is therefore consistent with the geomorphic zonation scheme first described from Grand Cayman by Blanchon & Jones (1995) and later summarized by Blanchon (2011), who both used slope breaks to identify reefal boundaries. We adopted this basic geomorphic scheme for all sectors of the MART including both Belize and Honduras.

In the northern Mexican sector of the MART, fringing reefs are relatively underdeveloped with limited reef-fronts that only extend to a depth of ∼6−8 m, thereby producing a more extensive bedrock terrace veneered by coral grounds (Blanchon et al., 2017). The edge of this terrace is marked by a slope break (mid-shelf break) and is commonly covered by a non-accretionary coral-ground community (Rodríguez-Martínez et al., 2011). In the Belize sector, fringing reefs transition into better developed Barriers and Atolls, and their reef-fronts extend into deeper water covering the bedrock terrace. At some sites, reef-front subzones like the spur-and-groove can extend all the way to the shelf edge in 25–30 m of water (James & Ginsburg, 1979; Burke, 1982). In the Honduras sector, reef development is restricted to the offshore Bay Island group: Roatan, Utila, Guanaja, and Cayos Cochinos. These reefs also have extensive spurs-and-groove zones extending to the shelf edge (Almada-Villela et al., 2003).

Following the geomorphic scheme of Blanchon (2011) we classify all sites based on retrospective satellite imagery from virtual globes such as Google Earth and ArcGIS online. A small number of sites (∼2%) have unclear images due to cloud cover and/or sea state, and geomorphic zoning is more difficult to delineate at the subzone level (for example, defining the boundary between the spur-and-grooves and non-tree bedrock terrace). In these few cases, we classify the sites as the most common type for that depth and zone based on field experience and expert opinion. Sites where reefs are less organized (∼17%) and lack a clear geomorphic structure, are simply classified as undifferentiated zones and assigned as either protected or exposed wave environments. Although uncommon, some of these ‘exposed’ zones can be found in semi-sheltered sites with complex coastal geography (*e.g*., the barrier reef fronts behind offshore atolls in the Belize sector).

### Coral distribution in geomorphic zones

To document the spatial distribution of scleractinian coral within shallow geomorphic zones we use information from two databases, the Caribbean Reef Information System (CRIS, Barcolab.org) and the Healthy Reefs Initiative (www.healthyreefs.org), which contain qualitative data on coral cover estimates at site level. Data in these sources come from published literature, research projects, and monitoring programs. In all cases, coral cover information was obtained using two similar benthic survey methodologies (Line Intercept Transect- LIT and Point Intercept Transect-PIT) according to Atlantic and Gulf Rapid Reef Assessment Benthic Protocol (AGRAA, Lang et al., 2012 available at https://www.agrra.org/coral-reef-monitoring) and the Mesoamerican Barrier Reef System monitoring program (Almada-Villela et al., 2003). Each survey includes between six and ten transects (10 or 30 m in length) haphazardly deployed at the reef sites. From these databases we select only the most recent data from sites in a 1–10 m depth range that contain coral cover estimates at species level. After discarding sites outside these requirements (*e.g*., depth range, data quality at species level), we compile a subset of coral coverage from 95 survey sites between 2016 and 2018 (Table 1). This time period is chosen to avoid potential bias caused by the recent outbreak of the Stony Coral Tissue Loss Disease reported in the Mexican sector during 2018 (Alvarez-Filip et al., 2019).

**Table 1:** Species richness (S), Diversity (H′) and abiotic factors (D, WE, HO) by geomorphic zone and environmental exposure. Abiotic factors correspond to physical environments of the Caribbean Sea stratification scheme of mechanical disturbances acting over coral communities at the surveyed reef sites. These include both chronic disturbances from wind-driven wave exposure and acute disturbances (Chollett et al., 2012). Chronic stress, given by wave exposure (WE), is related to wind conditions between 1999 and 2008 for the entire basin, whereas acute stress is given by the frequency of occurrence of hurricanes (HO) with Category 1–5 magnitudes in the last 157 years (1851–2008). Values of mechanical disturbances (*) are extracted from geographic information layers. D: average depth in meters, WE: Wave exposure in J m^−3^, HO: hurricane occurrence in number of events, SD: standard deviation.

Geomorphic zone	S ± SD	H′(av.) ± SD	D ± SD	WE* ± SD	HO* ± SD
Reef Front_sheltered	2.1 ± 1.2	0.5 ± 0.5	1.8 ± 2.9	5.9 ± 0.9	14 ± 2.1
Back Reef_sheltered	3.8 ± 2.0	1.0 ± 0.5	2.8 ± 2.9	7.5 ± 0.1	18 ± 4.7
Irregular_sheleted	3.8 ± 2.0	1.0 ± 0.5	2.9 ± 2.6	6.3 ± 1.4	11 ± 2.0
Irregular_exposed	3.8 ± 2.0	1.0 ± 0.5	3.7 ± 1.8	7.6 ± 0.1	20 ± 2.8
Reef Front_exposed	3.8 ± 2.0	1.0 ± 0.5	5.9 ± 2.5	7.1 ± 0.8	12 ± 5.4
Spur & Grooves_exposed	3.9 ± 2.0	1.0 ± 0.5	6.5 ± 2.5	7.3 ± 0.5	12 ± 1.1
Coral Ground_exposed	3.9 ± 2.0	1.0 ± 0.5	11.2 ± 4.2	7.4 ± 0.2	12 ± 4.7

**Figure 2:**
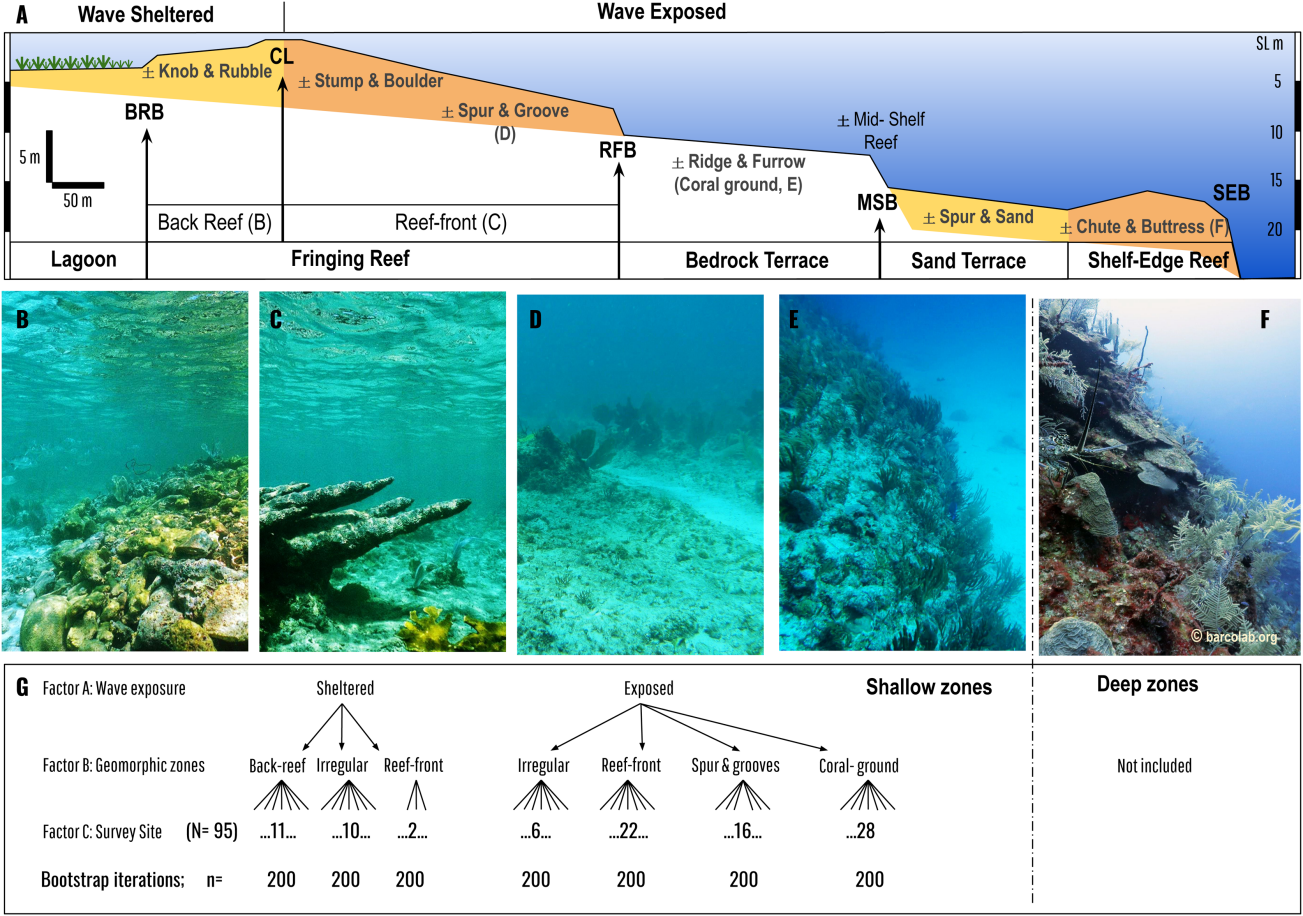
Geomorphic zonation of an idealized shelf showing shallow reef and benthic zones typical of Mesoamerican reef seascapes (following Blanchon et al., 2017). Zones are delineated by slope breaks: Back-Reef break (BRB), Crestline (CL), Reef-Front break (RFB), Mid-Shelf break (MSB), and Shelf-Edge break (SEB). Main benthic zones in bold are divided into wave sheltered (such as lagoon and back-reef) and wave exposed (such as reef-front, bedrock terrace, sand terrace *etc*.) (see Blanchon & Jones (1995) for detailed descriptions). Each benthic zone can be divided into sub-zones that may or maynot be present (±). Coloured zones are depositional or accretionary (like the fringing reef, or shelf-edge reef) whereas non-coloured are erosional or non-accretionary (like the bedrock terrace with its superficial coral ground). Our analysis is restricted to coral communities covering the fringing reef and the adjacent bedrock terrace (coral ground). Photos (B–F) give an overview of these coral communities: Back Reef (B), Reef Front (C), Spur & Groove (D), Coral-ground, and mid-shelf reef (E), and Shelf-Edge reef (F, which is not included in the study). (G) is a schematic of the asymmetrical design for PERMANOVA analysis encompassing three factors: wave exposure, geomorphic zones and survey site, with different levels of nested factors (back reef, reef front, irregular *etc*.) encompassed by 2 upper levels (sheltered and exposed).

Based on this subset, we calculate (i) the overall site live coral-cover data (LCC) and (ii) the relative coral cover of each species (*i.e*. the relative contribution of each species to the total coral cover). To avoid potential confusion stemming from using the term ‘coral cover’ to refer to the relative coral cover of each species, hereafter we refer to the latter as ‘coral species contribution’. In ecological studies, this is referred to as the sites taxonomic *β*-diversity and can be partitioned into two components: one reflecting species replacement between communities (turnover) and other reflecting the difference in the number of species among communities (nestedness-resultant; Baselga, 2012). However, coral-cover data are pooled and analyzed according to their geomorphic zonation, and not survey sites. Therefore, we compare overall coral-cover data of multiple sites as a comparative sampling unit, for example, pooling coral-cover from all back-reef sites (Fig. 2B). The qualitative *β*-diversity analysis entails partitioning the total dissimilarity in species composition (represented by the Sorensen index) into its turnover and nestedness components using 100 samples of 10 sites from each geomorphic zone. The analysis was conducted on Sorensen dissimilarity matrix using a betapart package available in R CRAN (Baselga et al., 2017).

### Statistical analysis

The statistical approach we use for the comparative analysis of benthic parameters and their geomorphological settings is similar to the method described by Medina-Valmaseda et al. (2020) who analyzed the geomorphic patterns of coral species at a single site, Punta Maroma. The main goal in using multivariate analysis in that study, and in this one, is to minimize method-bias of benthic surveys from multivariate-based analyses of ecological, abundance-based variables, which include a narrow set of pre-treatments and transforming of the raw data (*e.g*., standardization). For the multivariate pre-treatment, we choose a medium level (square-root) of data transformation. Further transformation is followed by an ordination process where we construct the correspondent Bray–Curtis matrix of similarities. All subsequent multivariate statistical analyses and graphical outputs are performed and constructed using Plymouth Routines in Multivariate Ecological Research (Primer-e version 7.0.13, serial number 4901, Clarke & Gorley, 2015).

To compare overall coral cover and related coral species contribution to relative cover of the communities between geomorphic zones, we conduct an asymmetrical analyses of permutational variance (PERMANOVA) at both levels of aggregation for factor ‘site’ (nested within the geomorphic zones by wave exposure) and nested in wave exposure regimes on the basis of Bray–Curtis similarity measures of transformed square-root matrix of ecological data (Anderson, 2006). Including these permutations, procedures offer an alternative to Normal-based inferential methods as they are adaptable and require fewer assumptions, whereas the asymmetrical design deals with a different number of geomorphic zones by wave exposure, with three sheltered and four exposed. The experimental design consists of three factors (Fig. 2C): Factor A: wave exposure (fixed with a = 2 levels: sheltered and exposed), Factor B: Geomorphic zones (random, nested in wave exposure with five levels: irregular, coral-ground, spur & grooves, reef-front and back reef), and Factor C: Site (random, nested in geomorphic zones, wave exposure) with 95 levels. The test uses permutation of residuals under a reduced model and Type III (partial Square Sums) in 9,999 permutations.

To test the homogeneity of multivariate data dispersion in each case, we performed a non-parametric permutational analysis of multivariate dispersion (PERMDISP), along with pairwise comparisons of the Bray–Curtis matrix of similarities. PERMDISP is performed based on distances to centroids, with *P*-values (p(perm)) obtained from 9999 permutations (Anderson, Ellingsen & McArdle, 2006). To determine the species contributions to coral cover within each geomorphic zone we conduct a two-way similarity percentage analysis (SIMPER) for zones by wave exposure, based on Bray–Curtis similarity measures of transformed square-root matrix of abundance data, making a 70% cut-off for low contributions (Clarke & Warwick, 1994; Clarke et al., 2014). The results are presented through the metric MDS of bootstrapped averages for 95% region estimates. Bootstrap averages test iteratively resample each group by its geomorphic zone 200 times, creating a plot of 4 m dimensions multivariate effect. Such a bootstraps procedure allows better visualization of how the mean response of each data group varies with changes in predictor values if we were to collect another sample of observations from a different set of geomorphic zones (Fieberg et al., 2009). The arrangement of the number of sites by its geomorphic zone classification lacks sampling balance (Fig. 2B) and therefore, another advantage of this approach is its success in estimating parameters of a statistical distribution for a balanced number of copies from our data whereas preserving the original structure of its data set (Fieberg, Vitense & Johnson, 2020).

### Extrinsic factors

For the purposes of this study, we consider extrinsic factors to be the diverse environmental variables that describe the abiotic context of coral communities including physicochemical variables and non-biotic disturbances, such as depth gradient, wave exposure and hurricane impacts. To evaluate the response of the biotic multivariate data to its corresponding multivariate environmental data we select information from both benthic surveys and cyclonic disturbances available for the MART. We use depth of benthic surveys and environmental information extracted from the open-source Physical Environments of the Caribbean Sea classification of Chollett et al. (2012) as geographic information system layers. For this, we extract data of physical disturbances including exposure to wind- and tropical-storm waves and the number of hurricane impacts using the QGIS (v. 3.14 Pi) Point Sampling Tool plugin, v.0.5.3 available at https://github.com/borysiasty/pointsamplingtool. Where the plugin failed because of no-value pixels, we manually selected the neighbouring major value. Furthermore, we check the distribution of all environmental variables for skewness and outliers and log-transform all data except the depth of each sampling site (following Clarke & Gorley, 2006). Finally, we normalize data to place each variable on the same dimensionless scale (for example, Clarke, Tweedley & Valesini, 2014). Environmental data are graphically represented through a Principal Coordinate Analysis (PCO) where vectors are the raw Pearson correlations of variables with the Principal Component Analysis (PCA) values (Torgerson, 1958; Gower, 1966).

To analyze and generate an exploratory hypothesis model on the spatial relationship between multivariate data clouds of the biotic response variables (coral species distribution) and that of regional environmental gradients, we test a distance-based linear model (DISTLM, Legendre & Anderson, 1999). The DISTLM test includes the Bray–Curtis matrices of previously square transformed data of coral species distribution and logarithmic Euclidean distance-based matrix of previously normalized environmental data in 9999 permutations within the factor Geomorphic zone for the environment. The test assumes non-linearity and additivity of the abiotic variables on the high-d community response. To visualize fitting models in multi-dimensional space we use the distance-based redundancy analysis (dbRDA, Legendre & Anderson, 1999; McArdle & Anderson, 2001) based on the Euclidean-distance matrix of environmental data.

## Results

### Coral cover patterns in geomorphic zones

**Figure 3:**
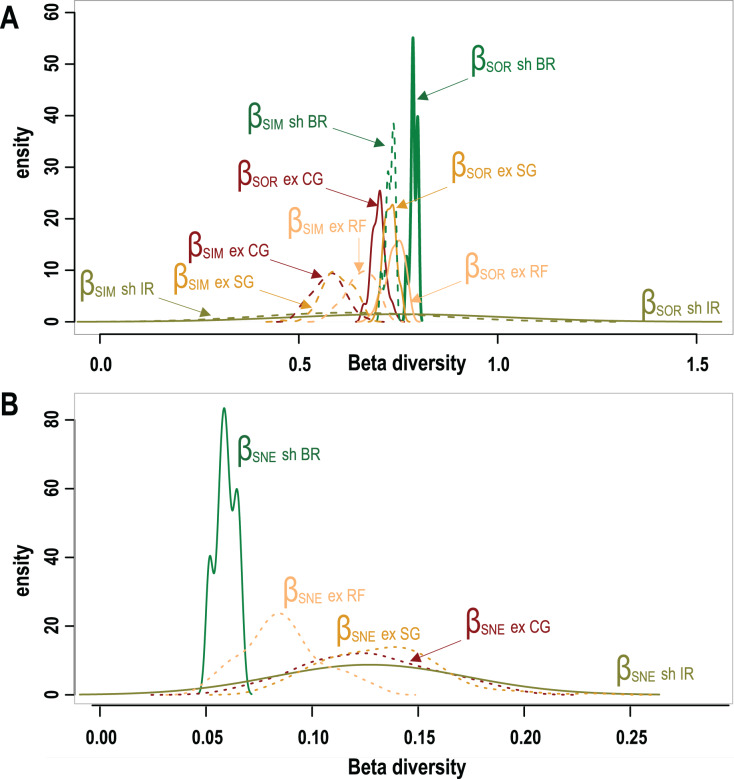
Taxonomic β-diversity of geomorphic zones and its components after Baselga (2012). (A) The overall β-diversity, measured as Sorensen dissimilarity β_SOR_ (solid lines) and values of the turnover component, measured as Simpson dissimilarity β_SIM_ (dashed lines). (B) Values of the nestedness-resultant fraction of the Sorensen dissimilarity β_SNE_. Note singular patterns in wave-sheltered back reef zones, and the similar patterns of all wave exposed zones (dotted lines) with that of the wave-sheltered irregular zone. Geomorphic zones by wave-exposure follow the colour code in Fig. 1: the prefixes “sh” and “ex” correspond to wave-sheltered and wave-exposed zones respectively. Zones are denoted by BR - back reef, IR - irregular zone, RF - reef front, SG - spur & grooves, CG - coral ground.

A total of 95 survey sites with 50 from Mexico, 25 from Belize and 20 sites from Honduras are included in this study. Sites encompass seven different types of geomorphic zones and subzones in both wave-exposed and protected environments along the regional latitudinal gradient and contain a total of 40 coral species. As shown in Fig. 3 results of *β*-diversity analysis show high levels of Total *β*-diversity (Fig. 3A, solid lines) for every geomorphic zone with high values and contribution of their turnover component (Fig. 3B, dashed lines) and low values for their nestedness-resultant component (Fig. 3B). Such results indicate that there is a low proportion of shared species between coral communities from adjacent geomorphic zones with a similar number of species. Although differences in taxonomic *β*-diversity values are clear between coral communities from sheltered back reef and exposed coral-ground zones, these are diffuse and overlapping for the communities from exposed reef front and spur & groove zones. The lower value of the nestedness-resultant fraction of Sorensen dissimilarity belongs to coral communities from sheltered back reef zones, whereas those from the irregular zones of wave-sheltered environments and wave-exposed spur & groove and coral-ground zones show almost identical patterns, indicating a confluence in these components for the exposed zones (Fig. 3B). Under the setting conditions of the *β*-diversity test, more sites were requested for sampling in the available datasets from two of the seven geomorphic zones. Results of the test are therefore unavailable for wave-exposed irregular zones (6 sampling sites), and wave-sheltered reef fronts (2 sampling sites).

**Figure 4:**
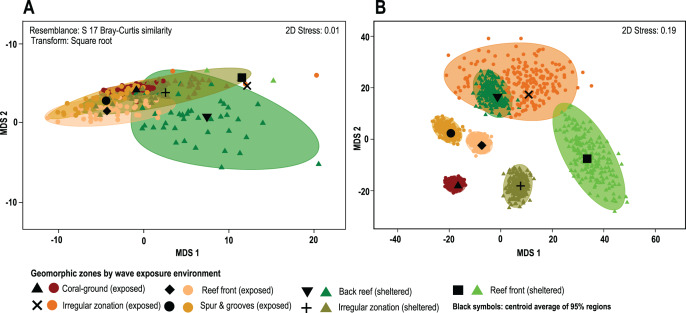
Coral cover patterns by geomorphic zone. (A) The similarity of overall coral cover for each zone type and wave exposure, using a metric MDS of ‘whole sample’ bootstrap averages resampling the n-transects of each sampling site 200 times (arranged by geomorphic zones and wave environment). nMDS shows the approximate 95% region estimates fitted to the bootstrap averages and the relative estimated position of centroids for each group (black symbols). (B) The species contribution to the relative cover within each geomorphic zone. The contribution of coral species to each geomorphic zone clearly represents long-term spatial patterns and highlights the absence of complete species homogenization across the reef seascape (3D stress: 0.08).

The results of the PERMANOVA test indicates that there are no significant differences in overall coral cover between wave sheltered and wave-exposed geomorphic zones (P(perm) = 0.39; Fig. 4A; Data S1), although when species identity is included in the relative composition analysis, there are significant differences between geomorphic zones (Fig. 4B). The results of the asymmetrical PERMANOVA test including the three terms ‘wave exposure’, ‘Geo zone (wave exposure)’ and ‘site (Geo zone (wave exposure))’ show that there are marginal differences among the wave exposure environments in assemblage structure (P(perm) = 0.06; P(MC) = 0.07) whereas there is a significant variability among assemblage structure from the geomorphic zones (P(perm) and P(MC) < 0.01, Fig. 4A; Data S2). The estimates of components of variation (S) for the three orthogonal factors are wave exposure: 115.82; Geo zone nested in wave exposure: 214.98; and site nested in Geo zone nested in wave exposure: 875.33. The site factor therefore contributes most to differences in species contribution, followed by the geomorphic zone, with wave exposure having the least control.

*A posteriori* pairwise PERMDISP test at the LCC level shows homogeneity of variance in all groups (F: 1.49, df1: 6, df2: 84; p(perm): 0.3422; Data S3), whereas the species contribution level shows heterogeneity in data variance involving pairwise tests for single or combined irregular and exposed zones (F: 13.23, df1: 6, df2: 543; p(perm): <0.01; Data S4). Other cases, including the irregular and combined sheltered/exposed geomorphic zones, show homogeneity in data variances (F: 13.23, df1: 6, df2: 543; p(perm): <0.01; Fig. 4B). In total 61% of possible pairwise tests show homogeneity in data variance and do not show a particular trend under the current PERMANOVA design. This indicates that there are differences in coral-species distribution between geomorphic zones (PERMANOVA), and in the variance of data distribution (PERMDISP) in several zones within the same environments (Fig. 4, Data S4).

The contribution of individual species to observed differences is assessed using the SIMPER test (Data S5). Results show that in exposed geomorphic zones three species overlap: *Agaricia agaricites, Porites astreoides* and *Siderastrea siderea* (Average similarity: 30.0) with relative contributions of 32.2%, 24.3% and 15.1% respectively. In sheltered zones another trio of species overlap, *P. astreoides, Orbicella annularis* and *Agaricia agaricites* (Average similarity 25.7) with relative contributions of 49.0%, 18.5% and 8.4% respectively (Data S1 to Data S5). Interestingly, an almost identical group of species accounts for the 81.2% average dissimilarity between samples. In terms of dissimilarity, only four species (*S. siderea, Porites astreoides, O. annularis* and *A. agaricites*), which contribute ∼10%, form roughly 53% of the differences between samples. Overall, across all wave exposure zones, the largest average similarity of 34.9% corresponds to the exposed Coral-ground zone, and the lowest 18.2% to the sheltered Back-reef zone.

### Coral species patterns and extrinsic factors

Average species richness (S, the number of species identified to lowest possible taxonomic level) increase with depth being the lowest in sheltered back-reef (2.1) and the highest in deeper coral-ground zone (4.9). Also species diversity as measured by the Shannon–Weiner index (H′) follows the same pattern, increasing seaward from the back- reef (0.9) towards coral-ground zone (1.4; Table 1).

**Figure 5:**
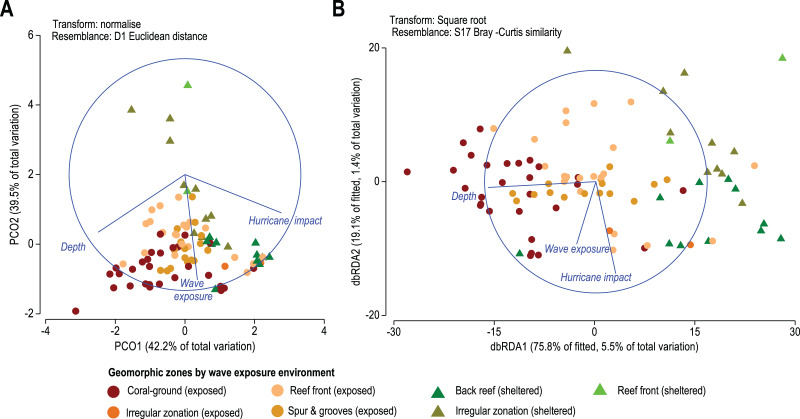
(A) Principal Coordinates Analysis (PCO) shows the multivariate environmental data cloud of three selected variables, depth, wave exposure and hurricane impact. Note the first two axes explain 81.6% of the internal variability of the three variables with depth contrasting with hurricane and depth and wave exposure acting differently. (B) Distance-based Redundancy Analysis (db-RDA) graphically illustrates the Distance-based linear model (DISTLM) results. It shows correlation trends between the Bray–Curtis distance matrix of biotic response and the explanatory Euclidean distance matrix of environmental variables.

On a regional scale, all sites are exposed to a similar wave-exposure regime regardless of geomorphic zone, whereas hurricane impact varies between sites (Fig. 5). When analyzed by geomorphic zone and wave exposure, the average site depth varies from 1.8 ± 2.9 m in sheltered reef-front zones, to 11.2 ± 4.2 m in exposed coral-ground zones. Chronic stress derived from wave-exposure for these sites varies between 5.9 ± 0.9 and 7.4 ± 0.2 J m^−3^. Whereas acute mechanical stress derived from hurricane frequency in the last 157 years (1851–2008) varies between 11 ± 2 years and 20 ± 2.8 years (Table 1). Regional patterns of three selected environmental data are presented in the Principal Coordinates Analysis (PCO) of Fig. 5. It shows that the first two axes explain 81.6% of the internal variability of these three environmental variables, indicating that the 2-d ordination is likely to have captured the majority of the patterns of multidimensional data variation. Accordingly, the depth of the reef site and hurricane impact explain 42.2% of variations of the PCO1 axis, and wave energy exposure alone explains 39.5% of the PCO2 axis. In addition, depth and hurricane impact appear to act inversely over coral species patterns (Fig. 5A).

Results from the DISTLM marginal tests (Data S6) show how much can be explained by each abiotic variable alone, ignoring other variables. Tests show that the depth of reef sites exerts the highest influence explaining 5.5% of the overall variability in coral species contribution (*p* < 0.01, Data S4A, Fig. 5B). Wave exposure and the number of major hurricane impacts when each is considered alone explain roughly 1% to 1.5% (*p* < 0.01, Data S4A, Fig. 5B) of variability in species contribution to each geomorphic zone. These results indicate that spatial patterns in coral species contribution by geomorphic zones are still responsive to these three environmental variables.

## Discussion

Our findings confirm the decline in acroporids on Mesoamerican reefs and the shift to an alternate ‘weedy’ state, but show that this has not yielded a complete homogenization of the coral community. Although data analysis shows that the overall coral cover of is similar between geomorphic zones, species patterns within them are statistically different. All zones are dominated by the same community of generalist species (*Agaricia and Porites*), but these are distributed differently in space. In exposed zones, *Agaricia agaricites* makes the largest contribution followed by *P. astreoides* and *Siderastrea siderea*, whereas in sheltered zones *Porites astreoides* dominates followed by *Orbicella annularis* and *A. agaricites*. The species responsible for differences between zones are *S. siderea, P. astreoides* and *O. annularis*. These differences confirm that environmental factors such as wave and hurricane exposure still have an impact on species distribution patterns, despite the large historical decline in coral cover.

When looking at similarities in the distribution of reef-building species, the Beta-diversity analysis provides a comprehensive overview of how it varies at the scales of tens to hundreds of meters encompassing the geomorphic framework of the MART. The comparison of compositional differences between geomorphic zones, further partitioned into turnover and nestedness after Baselga et al. (2017), allows further understanding of how coral biodiversity is assembled across wave-energy gradients of sheltered and exposed geomorphic zones. These results are consistent with previous studies indicating that changes in species composition at small scales occurs as a consequence of historical spatial constraints or environmental sorting, for example, in response to depth (*e.g*., Izsak & Price, 2001; Ellingsen & Gray, 2002). Similarly, other studies have also highlighted the relevance of environmental drivers in configuring local coral assemblages (Murdoch & Aronson, 1999; Williams et al., 2015a; Miyazawa et al., 2020). Our findings thus expand the comprehension of the factors underlying the turnover in species distribution in concordance with the geomorphic zonation.

We recognize that these factors generate a weak signal in the degraded coral community and this is likely impacted by the methodological protocol. For example, only a limited number of long-term variables were considered in analysing the species distribution patterns and, two of these (waves and hurricanes), exhibit significant variability over short temporal scales. Nevertheless, both have been reported as major environmental drivers of ecological processes including species zonation (Geister, 1977; Hughes et al., 2019). Moreover, the impact of such disturbances creates an adaptive biotic response by influencing the life-history traits of coral assemblages and creating scale-dependent patterns of species distribution, with some being better adapted to hurricane impacts (*e.g*., Chollett & Mumby, 2012). In addition, the result is also consistent with multiple reports of the increasingly important role that tropical cyclones play in shaping coral reef ecosystems at both short- and long-term scales (Blanchon, Jones & Kalbfleisch, 1997; Lugo, Rogers & Nixon, 2000; Blanchon et al., 2017; Hogan et al., 2020; Puotinen et al., 2020). Moreover, several schemes have proposed an adaptive response of coral assemblages to wave exposure regimes (*e.g*., Adey & Burke, 1977; Geister, 1977). To some extent, our results augment these schemes by assigning a geomorphic framework and providing an alternative approach to imprecisely defined species-zonation limits. However, wave-fetch becomes saturated on a regional scale and its role in these schemes has been questioned (Adey, 1978). If this is accurate then it could help explain the linkage weakness in Mesoamerican reefs.

The replacement of former acroporid-dominated zones by modern generalist assemblages may result from a species succession associated with hurricane disturbance, where an initial post-hurricane adjustment stage is combined with the loss of species redundancy (McWilliam et al., 2018). Generalist assemblages are formed by pioneer species which are considered to be adapted to repeated disturbance but controlled by depth-related parameters. The evolutionary success of *P. astreoides*, for example, is supported by its limited longevity and its weedy life-history strategy, which allows it to thrive in a wide variety of shallow habitats (Tomascik & Sander, 1987). In addition, pioneer assemblages that remain after disturbances are vestigial, given the large historical reductions in absolute abundance (which has dropped ∼50%) and loss in species redundancy (leaving only generalists). Consequently, some weakness is expected in the biotic-environmental signal and, with these data, it is not possible to rule-out a successional community status (González-Barrios, Cabral-Tena & Alvarez-Filip, 2021). The environmental variables may also act as a proxy for other unknown variables influencing coral assemblages (Ellis et al., 2019; Hughes et al., 2019).

The impact of reef geomorphology on coral species composition has been largely ignored in both original (local) and regional analyses (*e.g*., Rioja-Nieto & Álvarez-Filip, 2019; Contreras-Silva et al., 2020; Estrada-Saldí­var et al., 2019). In the case of local studies, that omission has less impact given that the environment is more homogeneous (but see Medina-Valmaseda et al., 2020), but becomes a problem at a regional scale as environment heterogeneity increases. Furthermore, many local studies pooled data from combined geomorphic zones such as the ‘fore-reef’ which lump non-accreting zones from the surrounding seascape with accretionary reef zones (Williams et al., 2015b; Medina-Valmaseda et al., 2020). By providing a more accurate geomorphology, our results clearly show a pattern of depth-related geomorphic control down the entire Mesoamerican reef tract, confirming that the geomorphic framework is a long-term result of the feedback between environmental processes and coral communities. A similar finding from another site in the region has been reported by Medina-Valmaseda et al. (2020) who showed that the inclusion of geomorphic zones in factor-analysis helped identify differences in coral species distribution patterns. Together, these findings underline the importance of depth-related geomorphic controls on large-scale coral-species patterns. Indeed, it is remarkable that geomorphic control prevails on coral communities, despite the long duration over which reef decline has occurred.

Geomorphic context was indirectly addressed by a recent province-wide meta-analysis (Jackson et al., 2014), which highlighted the significance of reef environment and depth, amongst others. The resulting trends, however, are still based on pooling of local data, and thereby relegate the role of environmental factors. Perhaps this lack of consideration of geomorphic context in ecological studies is a methodological artifact whereby reef environment and geomorphic context are relegated to the study site section. Regardless of the cause, the use of a detailed geomorphic zonation within any ecological analysis facilitates the consideration of extrinsic long-term factors. It also provides long-term accretion boundaries to benthic communities and thus incorporates geological models of reef development which are an important source of information on spatial and temporal heterogeneity of the seascape. As a consequence, although ecological studies provide a detailed snapshot of recent changes in coral communities, without a precise geomorphic context, they risk providing an inaccurate picture of both ecological and long-term reef development (Aronson & Precht, 1997; Bellwood et al., 2004; Bruckner, 2012).

## Conclusions

Despite a large decline in historical cover, the depauperate coral communities on fringing reefs of the Mesoamerican Reef Tract still show species-level differences between depth-related geomorphic zones. These spatial differences, however, are subtle and have been missed by previous ecological surveys, which have claimed that communities are homogenized and therefore represent an unnatural alternative state and compromise the accretion potential of future reef development. Although the decline of acroporid framebuilders has resulted in a partial homogenization between zones, it is still uncertain if the new ‘pioneer state’ is stable on the long timescales over which reef accretion occurs. Moreover, species-level differences in the distribution of pioneer species between geomorphic zones may be a response to their adaptive life-history traits after prolonged disturbance. The fact that these species are early colonizers therefore points towards a post-disturbance adjustment, and implies that this community may result from a successional failure induced by chronic anthropogenic disturbance (related to mass tourism along the Mayan Riviera). Nevertheless, the health status of local communities can vary on short-term scales and adapt to fluctuations in disturbances.

We conclude that an accurate analysis of spatial ecological trends in coral reefs requires a detailed geomorphic framework in order to identify subtle changes in communities at large spatial scales. If a geomorphic context is not provided, then a random selection of coral sites will under-represent the complexity in species patterns. Consequently, we suggest that including a geomorphic context is a fundamental prerequisite for accurately determining the signal of ecological changes on local, regional, and provincial scales. Finally, our results do not exclude the possibility of similar heterogeneous patterns in coral communities at other levels of organization, such as reef type.

## Supplemental Information

10.7717/peerj.12590/supp-1 Supplemental Information 1 Results of asymmetrical permutational MANOVA (PERMANOVA) at coral cover by site (LCC/site) level of aggregation for factor ‘site’ (nested within the geomorphic zones by wave exposure) and nested in wave exposure regimes on the basis of Bray–Curtis s. The experimental design consisted of 3 factors: Factor A: wave exposure (fixed with a = 2 levels: sheltered and exposed), Factor B: Geomorphic zones (random, nested in wave exposure with 5 levels), and Factor C: Site (random, nested in geomorphic zones, wave exposure) with 91 levels. The test uses permutation of residuals under a reduced model and Type III (partial Square Sums) in 9999 permutations.

10.7717/peerj.12590/supp-2 Supplemental Information 2 Results of asymmetrical permutational MANOVA (PERMANOVA) at coral species contribution to coral cover level of aggregation for factor ‘site’ (nested within the geomorphic zones by wave exposure) and nested in wave exposure regimes based on Bray–Curtis si. The experimental design consisted of 3 factors: Factor A: wave exposure (fixed with a = 2 levels: sheltered and exposed), Factor B: Geomorphic zones (random, nested in wave exposure with 5 levels), and Factor C: Site (random, nested in geomorphic zones, wave exposure) with 95 levels. The test uses permutation of residuals under a reduced model and Type III (partial Square Sums) in 9999 permutations.

10.7717/peerj.12590/supp-3 Supplemental Information 3 PERMDISP test results at coral cover by site level of aggregation. Distance-based test for homogeneity of multivariate dispersions. Zones by the wave exposure environment are abbreviate as follow: CG_exposed: Coral ground exposed, BR_sheletered: back reef sheltered; RF_sheltered/exposed: reef front sheltered and exposed respectively; Irregular_sheltered/exposed: lacking clear scheme of geomorphic zonation zones in sheltered and exposed to wave environments. P(perm): permutational p-value, t: statistic pseudo t.

10.7717/peerj.12590/supp-4 Supplemental Information 4 PERMDISP test results at species contribution to coral cover level of aggregation. Distance-based test for homogeneity of multivariate dispersions. Zones by environment are abbreviate as follow: CG_exposed: Coral ground exposed, BR_sheletered: back reef sheltered; RF_sheltered/exposed: reef front sheltered and exposed respectively; Irregular_sheltered/exposed: lacking of clear scheme of geomorphic zonation zones in sheltered and exposed to wave environments. P(perm): permutational p-value, t: statistic pseudo t.

10.7717/peerj.12590/supp-5 Supplemental Information 5 Two-way similarity percentage analysis (SIMPER) for zones by wave exposure, based on Bray–Curtis similarity measures of transformed square-root matrix of abundance data, making a 70% cut-off for low contributions. CG: Coral grounds, BR: back reef; RF: reef front sheltered and exposed respectively; Irregular: lacking of clear scheme of geomorphic zonation zones in sheltered and exposed to wave environments; S&G: spur and groove zone. Av.Abund: Average Abundance; Contrib%: spp. contribution in percentages; Cum.%: cumulative total (%) of contributions (70% cut-off); Av.Diss: average dissimilarities. SD: standard deviation of data.

10.7717/peerj.12590/supp-6 Supplemental Information 6 Distance-based linear model (DistLM) results.

## Additional Information and Declarations

### Data Availability

The following information was supplied regarding data availability: The raw data is available at GitHub: https://github.com/AlexisMedina2019/PeerJ-60115.
