# Supplementary material for: Geomorphically controlled coral distribution in degraded shallow reefs of the Western Caribbean
Source: PeerJ. 2022 Mar 14;10:e12590. doi: 10.7717/peerj.12590 (PMC8929170; doi:10.7717/peerj.12590)
Supplement: Supplemental Information 1 — The experimental design consisted of 3 factors: Factor A: wave exposure (fixed with a = 2 levels: sheltered and exposed), Factor B: Geomorphic zones (random, nested in wave exposure with 5 levels), and Factor C: Site (random, nested in geomorphic zones, wave exposure) with 91 levels. The test uses permutation of residuals under a reduced model and Type III (partial Square Sums) in 9999 permutations. [file peerj-10-12590-s001.docx]

**Supplemental Data S1.** Results of asymmetrical permutational MANOVA (PERMANOVA**) at coral cover by site (LCC/site) level of aggregation** for factor ‘site’ (nested within the geomorphic zones by wave exposure) and nested in wave exposure regimes on the basis of Bray-Curtis similarity measures of transformed square-root matrix of ecological data (Anderson, 2006). The experimental design consisted of 3 factors: Factor A: wave exposure (fixed with a = 2 levels: sheltered and exposed), Factor B: Geomorphic zones (random, nested in wave exposure with 5 levels: irregular zone, back-reef, reef front, spur & grooves and coral-grounds), and Factor C: Site (random, nested in geomorphic zones, wave exposure) with 91 levels. The test uses permutation of residuals under a reduced model and Type III (partial Square Sums) in 9999 permutations

*Resemblance worksheet*

Name: LCC by site

Data type: Similarity

Selection: All

Transform: Square root

Resemblance: S17 Bray-Curtis similarity

Sums of squares type: Type III (partial)

Fixed effects sum to zero for mixed terms

Permutation method: Permutation of residuals under a reduced model

Number of permutations: 9999

*Factors*

Name Abbrev. Type Levels

Environment En Fixed 2

Geo_zone Ge Random 5

site si Random 91

*PERMANOVA table of results*

Unique

Source df SS MS Pseudo-F P(perm) perms

En 1 362.96 362.96 1.9016 0.1622 9944

Ge(En) 5 975.94 195.19 1.04 0.3913 9938

si(Ge(En)) 84 15766 187.69 No test

Total 90 17863

*Details of the expected mean squares (EMS) for the model*

Source EMS

En 1*V(si(Ge(En))) + 4.9859*V(Ge(En)) + 17.094*S(En)

Ge(En) 1*V(si(Ge(En))) + 11.749*V(Ge(En))

si(Ge(En)) 1*V(si(Ge(En)))

*Construction of Pseudo-F ratio(s) from mean squares*

Source Numerator Denominator Num.df Den.df

En 1*En 0.42435*Ge(En) + 0.57565*si(Ge(En)) 1 24.11

Ge(En) 1*Ge(En) 1*si(Ge(En)) 5 84

si(Ge(En)) 0 0

*Estimates of components of variation*

Source Estimate Sq.root

S(En) 10.067 3.1729

V(Ge(En)) 0.63861 0.79913

V(si(Ge(En))) No test
