# Supplementary material for: Geomorphically controlled coral distribution in degraded shallow reefs of the Western Caribbean
Source: PeerJ. 2022 Mar 14;10:e12590. doi: 10.7717/peerj.12590 (PMC8929170; doi:10.7717/peerj.12590)
Supplement: Supplemental Information 2 — The experimental design consisted of 3 factors: Factor A: wave exposure (fixed with a = 2 levels: sheltered and exposed), Factor B: Geomorphic zones (random, nested in wave exposure with 5 levels), and Factor C: Site (random, nested in geomorphic zones, wave exposure) with 95 levels. The test uses permutation of residuals under a reduced model and Type III (partial Square Sums) in 9999 permutations. [file peerj-10-12590-s002.docx]

**Supplemental Data S2.** Results of asymmetrical permutational MANOVA (PERMANOVA**) at coral species contribution to coral cover level of aggregation** for factor ‘site’ (nested within the geomorphic zones by wave exposure) and nested in wave exposure regimes based on Bray-Curtis similarity measures of transformed square-root matrix of ecological data (Anderson, 2006). The experimental design consisted of 3 factors: Factor A: wave exposure (fixed with a = 2 levels: sheltered and exposed), Factor B: Geomorphic zones (random, nested in wave exposure with 5 levels: irregular zone, back-reef, reef front, spur & grooves and coral-grounds), and Factor C: Site (random, nested in geomorphic zones, wave exposure) with 95 levels. The test uses permutation of residuals under a reduced model and Type III (partial Square Sums) in 9999 permutations

Name: BC_corals_depurated

Data type: Similarity

Selection: All

Transform: Square root

Resemblance: S17 Bray-Curtis similarity

Sums of squares type: Type III (partial)

Fixed effects sum to zero for mixed terms

Permutation method: Permutation of residuals under a reduced model

Number of permutations: 9999

*Factors*

Name Abbrev. Type Levels

Environment En Fixed 2

Geo_zone Ge Random 5

site si Random 95

*PERMANOVA table of results*

Unique

Source df SS MS Pseudo-F P(perm) perms P(MC)

En 1 20708 20708 1.9078 0.0577 9932 0.0713

Ge(En) 5 1.0495E+05 20990 3.3409 0.0001 9862 0.0001

si(Ge(En)) 88 6.1682E+05 7009.3 3.6148 0.0001 9540 0.0001

Res 455 8.8226E+05 1939

Total 549 1.6614E+06

*Details of the expected mean squares (EMS) for the model*

Source EMS

En 1*V(Res) + 4.0907*V(si(Ge(En))) + 24.814*V(Ge(En)) + 85.078*S(En)

Ge(En) 1*V(Res) + 4.9623*V(si(Ge(En))) + 68.413*V(Ge(En))

si(Ge(En)) 1*V(Res) + 5.7924*V(si(Ge(En)))

Res 1*V(Res)

*Construction of Pseudo-F ratio(s) from mean squares*

Source Numerator Denominator Num.df Den.df

En 1*En 0.39548*si(Ge(En)) + 0.36271*Ge(En) + 0.24181*Res 1 10.09

Ge(En) 1*Ge(En) 0.85669*si(Ge(En)) + 0.14331*Res 5 96.29

si(Ge(En)) 1*si(Ge(En)) 1*Res 88 455

*Estimates of components of variation*

Source Estimate Sq.root

S(En) 115.82 10.762

V(Ge(En)) 214.98 14.662

V(si(Ge(En))) 875.33 29.586

V(Res) 1939 44.034
