# Supplementary material for: Geomorphically controlled coral distribution in degraded shallow reefs of the Western Caribbean
Source: PeerJ. 2022 Mar 14;10:e12590. doi: 10.7717/peerj.12590 (PMC8929170; doi:10.7717/peerj.12590)
Supplement: Supplemental Information 3 — Distance-based test for homogeneity of multivariate dispersions. Zones by the wave exposure environment are abbreviate as follow: CG_exposed: Coral ground exposed, BR_sheletered: back reef sheltered; RF_sheltered/exposed: reef front sheltered and exposed respectively; Irregular_sheltered/exposed: lacking clear scheme of geomorphic zonation zones in sheltered and exposed to wave environments. P(perm): permutational p-value, t: statistic pseudo t. [file peerj-10-12590-s003.docx]

**Data S4. PERMDISP test results at coral cover by site level of aggregation**. Distance-based test for homogeneity of multivariate dispersions. Zones by environment are abbreviate as follow: CG_exposed: Coral ground exposed, BR_sheletered: back reef sheltered; RF_sheltered/exposed: reef front sheltered and exposed respectively; Irregular_sheltered / exposed: lacking of clear scheme of geomorphic zonation zones in sheltered and exposed to wave environments. P(perm): permutational p-value, t: statistic pseudo t

*Resemblance worksheet*

Name: LCC by site

Data type: Similarity

Selection: All

Transform: Square root

Resemblance: S17 Bray-Curtis similarity

Group factor: Geozone_environment

Number of permutations: 9999

Number of groups: 7

Number of samples: 95

*DEVIATIONS FROM CENTROID*

F: 1.4975 df1: 6 df2: 88

P(perm): 0.3422

*PAIRWISE COMPARISONS*

Groups t P(perm)

(CG_exposed,BR_sheltered) 2.4199 0.0335

(CG_exposed,RF_sheltered) 1.079 0.4587

(CG_exposed,RF_exposed) 1.7349 0.144

(CG_exposed,S&G_exposed) 0.58297 0.5918

(CG_exposed,irregular_sheltered) 0.85895 0.4257

(CG_exposed,irregular_exposed) 0.047955 0.9652

(BR_sheltered,RF_sheltered) 1.2631 0.3167

(BR_sheltered,RF_exposed) 0.93158 0.4586

(BR_sheltered,S&G_exposed) 1.6507 0.1771

(BR_sheltered,irregular_sheltered) 1.1043 0.3666

(BR_sheltered,irregular_exposed) 0.74763 0.7254

(RF_sheltered,RF_exposed) 1.2873 0.3576

(RF_sheltered,S&G_exposed) 1.2579 0.3372

(RF_sheltered,irregular_sheltered) 1.1681 0.4533

(RF_exposed,S&G_exposed) 0.97868 0.4064

(RF_exposed,irregular_sheltered) 0.49268 0.6661

(RF_exposed,irregular_exposed) 0.53667 0.7635

(S&G_exposed,irregular_sheltered) 0.33718 0.7601

(S&G_exposed,irregular_exposed) 0.19846 0.9129

(irregular_sheltered,irregular_exposed) 0.32214 0.7982

*MEANS AND STANDARD ERRORS*

Group Size Average SE

CG_exposed 28 8.4784 1.1017

BR_sheltered 11 15.528 3.7682

RF_sheltered 2 3.9572 0

RF_exposed 22 12.055 1.8587

S&G_exposed 16 9.5639 1.5345

irregular_sheltered 10 10.478 2.4021

irregular_exposed 6 8.6793 1.0912
