# Supplementary material for: Geomorphically controlled coral distribution in degraded shallow reefs of the Western Caribbean
Source: PeerJ. 2022 Mar 14;10:e12590. doi: 10.7717/peerj.12590 (PMC8929170; doi:10.7717/peerj.12590)
Supplement: Supplemental Information 4 — Distance-based test for homogeneity of multivariate dispersions. Zones by environment are abbreviate as follow: CG_exposed: Coral ground exposed, BR_sheletered: back reef sheltered; RF_sheltered/exposed: reef front sheltered and exposed respectively; Irregular_sheltered/exposed: lacking of clear scheme of geomorphic zonation zones in sheltered and exposed to wave environments. P(perm): permutational p-value, t: statistic pseudo t. [file peerj-10-12590-s004.docx]

**Data S4. PERMDISP test results at species contribution to coral cover level of aggregation**. Distance-based test for homogeneity of multivariate dispersions. Zones by environment are abbreviate as follow: CG_exposed: Coral ground exposed, BR_sheletered: back reef sheltered; RF_sheltered/exposed: reef front sheltered and exposed respectively; Irregular_sheltered / exposed: lacking of clear scheme of geomorphic zonation zones in sheltered and exposed to wave environments. P(perm): permutational p-value, t: statistic pseudo t

*Resemblance worksheet*

Name: BC_corals_depurated

Data type: Similarity

Selection: All

Transform: Square root

Resemblance: S17 Bray-Curtis similarity

Group factor: Geozone_environment

Number of permutations: 9999

Number of groups: 7

Number of samples: 550

*DEVIATIONS FROM CENTROID*

F: 13.23 df1: 6 df2: 543

P(perm): 0.0001

*PAIRWISE COMPARISONS*

Groups t P(perm)

(CG_exposed,BR_sheltered) 6.8297 0.0001

(CG_exposed,RF_sheltered) 0.52265 0.6587

(CG_exposed,RF_exposed) 6.8312 0.0001

(CG_exposed,S&G_exposed) 2.8711 0.0085

(CG_exposed,irregular_sheltered) 0.3465 0.7508

(CG_exposed,irregular_exposed) 1.3032 0.2768

(BR_sheltered,RF_sheltered) 2.8299 0.0234

(BR_sheltered,RF_exposed) 1.7799 0.1152

(BR_sheltered,S&G_exposed) 4.2015 0.0004

(BR_sheltered,irregular_sheltered) 5.2052 0.0001

(BR_sheltered,irregular_exposed) 1.8683 0.1485

(RF_sheltered,RF_exposed) 2.1457 0.1065

(RF_sheltered,S&G_exposed) 0.70079 0.5723

(RF_sheltered,irregular_sheltered) 0.31292 0.7791

(RF_sheltered,irregular_exposed) 0.5784 0.6026

(RF_exposed,S&G_exposed) 3.2032 0.0028

(RF_exposed,irregular_sheltered) 4.6628 0.0002

(RF_exposed,irregular_exposed) 1.1708 0.3613

(S&G_exposed,irregular_sheltered) 1.8691 0.08

(S&G_exposed,irregular_exposed) 0.16809 0.8948

(irregular_sheltered,irregular_exposed) 1.0208 0.3741

*MEANS AND STANDARD ERRORS*

Group Size Average SE

CG_exposed 173 45.989 0.93293

BR_sheltered 61 58.289 1.482

RF_sheltered 12 47.9 3.4341

RF_exposed 139 55.188 0.95336

S&G_exposed 96 50.37 1.1706

irregular_sheltered 58 46.641 1.6844

irregular_exposed 11 50.997 4.1549
