# Supplementary material for: Geomorphically controlled coral distribution in degraded shallow reefs of the Western Caribbean
Source: PeerJ. 2022 Mar 14;10:e12590. doi: 10.7717/peerj.12590 (PMC8929170; doi:10.7717/peerj.12590)
Supplement: Supplemental Information 5 — CG: Coral grounds, BR: back reef; RF: reef front sheltered and exposed respectively; Irregular: lacking of clear scheme of geomorphic zonation zones in sheltered and exposed to wave environments; S&G: spur and groove zone. Av.Abund: Average Abundance; Contrib%: spp. contribution in percentages; Cum.%: cumulative total (%) of contributions (70% cut-off); Av.Diss: average dissimilarities. SD: standard deviation of data. [file peerj-10-12590-s005.docx]

**Supplemental Data S5.** **Two-way similarity percentage analysis (SIMPER)** for zones by wave exposure, based on Bray-Curtis similarity measures of transformed square-root matrix of abundance data, making a 70 % cut-off for low contributions. CG: Coral grounds, BR: back reef; RF: reef front sheltered and exposed respectively; Irregular: lacking of clear scheme of geomorphic zonation zones in sheltered and exposed to wave environments; S&G: spur and groove zone. Av.Abund: Average Abundance; Contrib%: spp. contribution in percentages; Cum.%: cumulative total (%) of contributions (70% cut-off); Av.Diss: average dissimilarities. SD: standard deviation of data.

Similarity Percentages - species contributions

Two-Way Analysis

*Data worksheet*

Name: SQRT_corals_depurated

Data type: Abundance

Sample selection: All

Variable selection: All

*Parameters*

Resemblance: S17 Bray-Curtis similarity

Cut off for low contributions: 70.00%

*Examines Environment groups*

*(across all Geo_zone groups)*

*Group exposed*

Average similarity: 30.01

Species Av.Abund Av.Sim Sim/SD Contrib% Cum.%

Agaricia agaricites 1.20 9.65 0.88 32.18 32.18

Porites astreoides 1.03 7.28 0.70 24.26 56.44

Siderastrea siderea 0.78 4.52 0.53 15.06 71.50

*Group sheltered*

Average similarity: 25.74

Species Av.Abund Av.Sim Sim/SD Contrib% Cum.%

Porites astreoides 1.10 12.62 0.87 49.03 49.03

Orbicella annularis 0.78 4.76 0.45 18.48 67.51

Agaricia agaricites 0.50 2.16 0.33 8.39 75.90

*Groups exposed & sheltered*

Average dissimilarity = 81.23

Group exposed Group sheltered

Species Av.Abund Av.Abund Av.Diss Diss/SD Contrib% Cum.%

Siderastrea siderea 0.78 0.51 13.58 1.00 16.72 16.72

Porites astreoides 1.03 1.10 10.61 0.98 13.06 29.77

Orbicella annularis 0.44 0.78 10.52 0.92 12.95 42.73

Agaricia agaricites 1.20 0.50 8.45 0.90 10.40 53.13

Agaricia tenuifolia 0.86 0.32 7.81 0.50 9.61 62.74

Porites porites 0.31 0.41 5.54 0.54 6.82 69.55

Pseudodiploria strigosa 0.41 0.24 4.82 0.70 5.93 75.48

*Examines Geo_zone groups*

*(across all Environment groups)*

*Group CG*

Average similarity: 34.89

Species Av.Abund Av.Sim Sim/SD Contrib% Cum.%

Agaricia agaricites 1.58 13.73 1.22 39.36 39.36

Porites astreoides 1.03 6.99 0.81 20.05 59.41

Siderastrea siderea 0.96 6.48 0.67 18.57 77.98

*Group BR*

Average similarity: 18.21

Species Av.Abund Av.Sim Sim/SD Contrib% Cum.%

Porites astreoides 0.92 11.44 0.72 62.80 62.80

Acropora palmata 0.76 2.11 0.21 11.60 74.40

*Group RF*

Average similarity: 22.80

Species Av.Abund Av.Sim Sim/SD Contrib% Cum.%

Porites astreoides 1.03 7.89 0.62 34.61 34.61

Agaricia agaricites 0.87 5.03 0.59 22.05 56.66

Agaricia tenuifolia 0.94 2.67 0.26 11.73 68.39

Siderastrea siderea 0.77 2.54 0.37 11.15 79.54

*Group S&G*

Average similarity: 29.44

Species Av.Abund Av.Sim Sim/SD Contrib% Cum.%

Agaricia tenuifolia 1.70 9.52 0.58 32.34 32.34

Porites astreoides 0.99 6.84 0.68 23.25 55.59

Agaricia agaricites 0.97 6.09 0.64 20.70 76.29

*Group irregular*

Average similarity: 33.54

Species Av.Abund Av.Sim Sim/SD Contrib% Cum.%

Porites astreoides 1.34 14.15 1.12 42.18 42.18

Orbicella annularis 1.29 9.68 0.73 28.86 71.04

*Groups CG & BR*

No pairs of groups with samples

*Groups CG & RF*

Average dissimilarity = 74.57

Group CG Group RF

Species Av.Abund Av.Abund Av.Diss Diss/SD Contrib% Cum.%

Agaricia agaricites 1.58 0.87 9.43 1.19 12.64 12.64

Porites astreoides 1.03 1.03 8.09 1.06 10.84 23.48

Siderastrea siderea 0.96 0.77 7.90 1.03 10.59 34.08

Agaricia tenuifolia 0.30 0.94 7.89 0.61 10.58 44.66

Orbicella faveolata 0.93 0.36 7.05 0.80 9.45 54.11

Orbicella annularis 0.33 0.59 5.00 0.65 6.71 60.81

Pseudodiploria strigosa 0.37 0.38 4.10 0.71 5.50 66.31

Porites porites 0.31 0.38 3.97 0.57 5.33 71.64

*Groups BR & RF*

Average dissimilarity = 86.26

Group BR Group RF

Species Av.Abund Av.Abund Av.Diss Diss/SD Contrib% Cum.%

Siderastrea siderea 0.20 0.77 21.54 1.22 24.98 24.98

Porites astreoides 0.92 1.03 12.11 0.98 14.04 39.02

Orbicella annularis 0.09 0.59 10.08 0.77 11.69 50.71

Acropora palmata 0.76 0.29 8.38 0.48 9.72 60.43

Porites porites 0.30 0.38 6.17 0.49 7.16 67.58

Pseudodiploria strigosa 0.33 0.38 6.15 0.53 7.13 74.71

*Groups CG & S&G*

Average dissimilarity = 73.07

Group CG Group S&G

Species Av.Abund Av.Abund Av.Diss Diss/SD Contrib% Cum.%

Agaricia tenuifolia 0.30 1.70 11.85 0.94 16.22 16.22

Agaricia agaricites 1.58 0.97 8.86 1.20 12.13 28.35

Porites astreoides 1.03 0.99 7.23 1.13 9.90 38.25

Siderastrea siderea 0.96 0.61 7.19 1.02 9.84 48.09

Orbicella faveolata 0.93 0.47 7.11 0.85 9.73 57.82

Pseudodiploria strigosa 0.37 0.51 4.97 0.73 6.80 64.62

Orbicella annularis 0.33 0.48 4.56 0.60 6.24 70.87

*Groups BR & S&G*

No pairs of groups with samples

*Groups RF & S&G*

Average dissimilarity = 75.07

Group RF Group S&G

Species Av.Abund Av.Abund Av.Diss Diss/SD Contrib% Cum.%

Agaricia tenuifolia 0.94 1.70 14.83 0.98 19.76 19.76

Porites astreoides 1.03 0.99 8.90 1.03 11.86 31.62

Agaricia agaricites 0.87 0.97 8.34 1.05 11.11 42.73

Siderastrea siderea 0.77 0.61 6.70 0.90 8.92 51.65

Orbicella annularis 0.59 0.48 6.03 0.69 8.03 59.69

Pseudodiploria strigosa 0.38 0.51 5.28 0.71 7.03 66.72

Orbicella faveolata 0.36 0.47 4.95 0.59 6.59 73.31

*Groups CG & irregular*

Average dissimilarity = 75.79

Group CG Group irregular

Species Av.Abund Av.Abund Av.Diss Diss/SD Contrib% Cum.%

Agaricia agaricites 1.58 0.78 11.76 1.28 15.51 15.51

Siderastrea siderea 0.96 0.57 8.56 1.06 11.29 26.80

Porites astreoides 1.03 1.34 7.60 1.12 10.03 36.84

Orbicella faveolata 0.93 0.10 7.31 0.79 9.64 46.48

Pseudodiploria clivosa 0.02 0.14 5.83 0.70 7.70 54.18

Pseudodiploria strigosa 0.37 0.16 5.10 0.80 6.73 60.91

Agaricia tenuifolia 0.30 0.24 3.86 0.59 5.09 66.00

Montastraea cavernosa 0.42 0.07 3.35 0.59 4.42 70.43

*Groups BR & irregular*

Average dissimilarity = 80.96

Group BR Group irregular

Species Av.Abund Av.Abund Av.Diss Diss/SD Contrib% Cum.%

Orbicella annularis 0.09 1.29 13.91 1.13 17.18 17.18

Porites astreoides 0.92 1.34 11.60 1.11 14.33 31.51

Agaricia agaricites 0.28 0.78 7.98 0.90 9.85 41.37

Siderastrea siderea 0.20 0.57 7.61 0.68 9.40 50.77

Porites porites 0.30 0.48 7.07 0.73 8.73 59.50

Acropora palmata 0.76 0.09 6.97 0.49 8.61 68.10

Agaricia tenuifolia 0.45 0.24 5.21 0.51 6.44 74.54

*Groups RF & irregular*

Average dissimilarity = 78.23

Group RF Group irregular

Species Av.Abund Av.Abund Av.Diss Diss/SD Contrib% Cum.%

Porites astreoides 1.03 1.34 10.75 1.05 13.75 13.75

Siderastrea siderea 0.77 0.57 10.46 0.95 13.37 27.11

Agaricia agaricites 0.87 0.78 8.34 0.94 10.66 37.77

Orbicella annularis 0.59 1.29 8.07 0.74 10.32 48.09

Agaricia tenuifolia 0.94 0.24 7.58 0.53 9.69 57.79

Porites porites 0.38 0.48 4.95 0.54 6.33 64.11

Pseudodiploria strigosa 0.38 0.16 4.78 0.71 6.11 70.23

*Groups S&G & irregular*

Average dissimilarity = 77.87

Group S&G Group irregular

Species Av.Abund Av.Abund Av.Diss Diss/SD Contrib% Cum.%

Agaricia tenuifolia 1.70 0.24 15.26 0.98 19.60 19.60

Agaricia agaricites 0.97 0.78 9.10 1.03 11.69 31.29

Porites astreoides 0.99 1.34 8.70 1.04 11.18 42.47

Siderastrea siderea 0.61 0.57 7.14 0.98 9.17 51.63

Pseudodiploria strigosa 0.51 0.16 6.59 0.78 8.47 60.10

Pseudodiploria clivosa 0.00 0.14 6.18 0.68 7.94 68.04

Orbicella annularis 0.48 1.29 4.62 0.54 5.93 73.97
