# Supplementary material for: Geomorphically controlled coral distribution in degraded shallow reefs of the Western Caribbean
Source: PeerJ. 2022 Mar 14;10:e12590. doi: 10.7717/peerj.12590 (PMC8929170; doi:10.7717/peerj.12590)
Supplement: Supplemental Information 6 [file peerj-10-12590-s006.docx]

**Supplemental Data S6. Distance-based linear model (DistLM) results**

Distance based linear models

*Resemblance worksheet*

Name: bioticdistlm

Data type: Similarity

Selection: All

Transform: Square root

Resemblance: S17 Bray-Curtis similarity

*Predictor variables worksheet*

Name: PhysFULLlog+1normal

Data type: Environmental

Sample selection: All

Variable selection: All

Transform: Log(X+1)

Normalise

Selection criterion: R^2

Selection procedure: Best

*VARIABLES*

1 Depth Trial

2 Wave_exposure Trial

3 Hurricane_impact Trial

Total SS(trace): 1.6614E+06

*MARGINAL TESTS*

Variable SS(trace) Pseudo-F P Prop.

Depth 91234 31.841 0.001 0.054914

Wave_exposure 17974 5.9935 0.001 0.010819

Hurricane_impact 26348 8.8306 0.001 0.015859

res.df: 548

NO STARTING TERMS

*BEST SOLUTIONS*

1 VARIABLE

R^2 RSS No.Vars Selections

0.0549141.5702E+06 1 1

0.0158591.6351E+06 1 3

0.0108191.6434E+06 1 2

2 VARIABLES

R^2 RSS No.Vars Selections

0.0668221.5504E+06 2 1,3

0.063429 1.556E+06 2 1,2

0.0265521.6173E+06 2 2,3

3 VARIABLES

R^2 RSS No.Vars Selections

0.0731131.5399E+06 3 All

BEST RESULT FOR EACH NUMBER OF VARIABLES

R^2 RSS No.Vars Selections

0.0549141.5702E+06 1 1

0.0668221.5504E+06 2 1,3

0.0731131.5399E+06 3 All

*OVERALL BEST SOLUTIONS*

R^2 RSS No.Vars Selections

0.0731131.5399E+06 3 All

0.0668221.5504E+06 2 1,3

0.063429 1.556E+06 2 1,2

0.0549141.5702E+06 1 1

0.0265521.6173E+06 2 2,3

0.0158591.6351E+06 1 3

0.0108191.6434E+06 1 2

*Percentage of variation explained by individual axes*

% explained variation % explained variation

out of fitted model out of total variation

Axis Individual Cumulative Individual Cumulative

1 75.79 75.79 5.54 5.54

2 19.11 94.9 1.4 6.94

3 5.1 100 0.37 7.31

*dbRDA coordinate scores*

Sample dbRDA1 dbRDA2 dbRDA3

1032B 01 -17.107 7.8801 4.8286

1032B 02 -17.107 7.8801 4.8286

1032B 03 -17.107 7.8801 4.8286

1032B 04 -17.107 7.8801 4.8286

1032B 05 -17.107 7.8801 4.8286

1032B 06 -17.107 7.8801 4.8286

40_Cannons_1 -2.4597 0.62789 1.5952

40_Cannons_2 -2.4597 0.62789 1.5952

40_Cannons_3 -2.4597 0.62789 1.5952

40_Cannons_4 -2.4597 0.62789 1.5952

40_Cannons_5 -2.4597 0.62789 1.5952

40_Cannons_6 -2.4597 0.62789 1.5952

Bocana Caricomp 1 -9.4369 -10.704 -4.1062

Bocana Caricomp 2 -9.4369 -10.704 -4.1062

Bocana Caricomp 3 -9.4369 -10.704 -4.1062

Bocana Caricomp 4 -9.4369 -10.704 -4.1062

Bocana Caricomp 5 -9.4369 -10.704 -4.1062

Bocana Caricomp 6 -9.4369 -10.704 -4.1062

Bonanza 1 27.867 -8.5453 0.51357

Bonanza 2 27.867 -8.5453 0.51357

Bonanza 3 27.867 -8.5453 0.51357

Bonanza 4 27.867 -8.5453 0.51357

Bonanza 5 27.867 -8.5453 0.51357

Bonanza Profundo 1 -8.3991 -10.97 -3.6215

Bonanza Profundo 2 -8.3991 -10.97 -3.6215

Bonanza Profundo 3 -8.3991 -10.97 -3.6215

Bonanza Profundo 4 -8.3991 -10.97 -3.6215

Bonanza Profundo 5 -8.3991 -10.97 -3.6215

Bonanza Profundo 6 -8.3991 -10.97 -3.6215

BZ1037A 1 11.285 6.0637 -0.66533

BZ1037A 2 11.285 6.0637 -0.66533

BZ1037A 3 11.285 6.0637 -0.66533

BZ1037A 4 11.285 6.0637 -0.66533

BZ1037A 5 11.285 6.0637 -0.66533

BZ1037A 6 11.285 6.0637 -0.66533

BZ1043A 1 28.094 18.447 -12.118

BZ1043A 2 28.094 18.447 -12.118

BZ1043A 3 28.094 18.447 -12.118

BZ1043A 4 28.094 18.447 -12.118

BZ1043A 5 28.094 18.447 -12.118

BZ1043A 6 28.094 18.447 -12.118

BZ1044 1 -2.1092 -1.4764 -0.014135

BZ1044 2 -2.1092 -1.4764 -0.014135

BZ1044 3 -2.1092 -1.4764 -0.014135

BZ1044 4 -2.1092 -1.4764 -0.014135

BZ1044 5 -2.1092 -1.4764 -0.014135

BZ1044 6 -2.1092 -1.4764 -0.014135

BZ1045 1 -6.8772 -0.31951 -3.9425

BZ1045 2 -6.8772 -0.31951 -3.9425

BZ1045 3 -6.8772 -0.31951 -3.9425

BZ1045 4 -6.8772 -0.31951 -3.9425

BZ1045 5 -6.8772 -0.31951 -3.9425

BZ1045 6 -6.8772 -0.31951 -3.9425

BZ1052 1 0.95579 0.36717 2.5178

BZ1052 2 0.95579 0.36717 2.5178

BZ1052 3 0.95579 0.36717 2.5178

BZ1052 4 0.95579 0.36717 2.5178

BZ1052 5 0.95579 0.36717 2.5178

BZ1052 6 0.95579 0.36717 2.5178

BZ1076B 1 -17.005 -3.3541 -0.75146

BZ1076B 2 -17.005 -3.3541 -0.75146

BZ1076B 3 -17.005 -3.3541 -0.75146

BZ1076B 4 -17.005 -3.3541 -0.75146

BZ1076B 5 -17.005 -3.3541 -0.75146

BZ1076B 6 -17.005 -3.3541 -0.75146

BZ1100 1 11.344 7.2857 -0.84186

BZ1100 2 11.344 7.2857 -0.84186

BZ1100 3 11.344 7.2857 -0.84186

BZ1100 4 11.344 7.2857 -0.84186

BZ1100 5 11.344 7.2857 -0.84186

BZ1100 6 11.344 7.2857 -0.84186

BZ1104 1 18.328 1.8652 2.0713

BZ1104 2 18.328 1.8652 2.0713

BZ1104 3 18.328 1.8652 2.0713

BZ1104 5 18.328 1.8652 2.0713

BZ1104 6 18.328 1.8652 2.0713

BZ1106 1 19.06 1.1369 1.0941

BZ1106 2 19.06 1.1369 1.0941

BZ1106 3 19.06 1.1369 1.0941

BZ1106 4 19.06 1.1369 1.0941

BZ1106 5 19.06 1.1369 1.0941

BZ1106 6 19.06 1.1369 1.0941

BZ1109 1 16.901 1.3789 4.3954

BZ1109 2 16.901 1.3789 4.3954

BZ1109 3 16.901 1.3789 4.3954

BZ1109 4 16.901 1.3789 4.3954

BZ1109 5 16.901 1.3789 4.3954

BZ1109 6 16.901 1.3789 4.3954

BZ1112 1 13.437 16.23 -7.7468

BZ1112 2 13.437 16.23 -7.7468

BZ1112 3 13.437 16.23 -7.7468

BZ1112 4 13.437 16.23 -7.7468

BZ1112 5 13.437 16.23 -7.7468

BZ1112 6 13.437 16.23 -7.7468

BZ1114 1 20.891 4.4629 6.4732

BZ1114 2 20.891 4.4629 6.4732

BZ1114 3 20.891 4.4629 6.4732

BZ1114 4 20.891 4.4629 6.4732

BZ1114 5 20.891 4.4629 6.4732

BZ1177 1 10.277 13.505 -5.3663

BZ1177 2 10.277 13.505 -5.3663

BZ1177 3 10.277 13.505 -5.3663

BZ1177 4 10.277 13.505 -5.3663

BZ1177 5 10.277 13.505 -5.3663

BZ1177 6 10.277 13.505 -5.3663

BZ1192 1 17.554 5.7594 -0.25748

BZ1192 2 17.554 5.7594 -0.25748

BZ1192 3 17.554 5.7594 -0.25748

BZ1192 4 17.554 5.7594 -0.25748

BZ1192 5 17.554 5.7594 -0.25748

BZ1192 6 17.554 5.7594 -0.25748

BZ1223 1 22.092 -3.2499 3.0047

BZ1223 2 22.092 -3.2499 3.0047

BZ1223 3 22.092 -3.2499 3.0047

BZ1223 4 22.092 -3.2499 3.0047

BZ1223 5 22.092 -3.2499 3.0047

BZ1223 6 22.092 -3.2499 3.0047

BZ1229 1 9.803 -2.4202 1.465

BZ1229 2 9.803 -2.4202 1.465

BZ1229 3 9.803 -2.4202 1.465

BZ1229 4 9.803 -2.4202 1.465

BZ1229 5 9.803 -2.4202 1.465

BZ1229 6 9.803 -2.4202 1.465

BZ1230 1 13.246 -1.3304 2.6968

BZ1230 2 13.246 -1.3304 2.6968

BZ1230 3 13.246 -1.3304 2.6968

BZ1230 4 13.246 -1.3304 2.6968

BZ1230 5 13.246 -1.3304 2.6968

BZ1230 6 13.246 -1.3304 2.6968

BZ1231 1 20.008 0.34077 4.0469

BZ1231 2 20.008 0.34077 4.0469

BZ1231 3 20.008 0.34077 4.0469

BZ1231 4 20.008 0.34077 4.0469

BZ1231 5 20.008 0.34077 4.0469

BZ1231 6 20.008 0.34077 4.0469

BZ1234 2 21.078 -1.1099 3.9204

BZ1234 3 21.078 -1.1099 3.9204

BZ1234 5 21.078 -1.1099 3.9204

BZ1234 6 21.078 -1.1099 3.9204

BZHMCwall 1 -4.0736 19.543 -14.631

BZHMCwall 2 -4.0736 19.543 -14.631

BZHMCwall 3 -4.0736 19.543 -14.631

BZHMCwall 4 -4.0736 19.543 -14.631

BZHMCwall 5 -4.0736 19.543 -14.631

BZHMCwall 6 -4.0736 19.543 -14.631

Canones 01 -15.713 -4.4162 -1.1285

Canones 02 -15.713 -4.4162 -1.1285

Canones 03 -15.713 -4.4162 -1.1285

Canones 04 -15.713 -4.4162 -1.1285

Canones 05 -15.713 -4.4162 -1.1285

Canones 06 -15.713 -4.4162 -1.1285

Casa Cenote 01 -7.4135 2.299 -1.1523

Casa Cenote 02 -7.4135 2.299 -1.1523

Casa Cenote 03 -7.4135 2.299 -1.1523

Casa Cenote 04 -7.4135 2.299 -1.1523

Casa Cenote 05 -7.4135 2.299 -1.1523

Casa Cenote 06 -7.4135 2.299 -1.1523

Cordilleras 01 -15.09 7.9557 5.1232

Cordilleras 02 -15.09 7.9557 5.1232

Cordilleras 03 -15.09 7.9557 5.1232

Cordilleras 04 -15.09 7.9557 5.1232

Cordilleras 05 -15.09 7.9557 5.1232

Cordilleras 06 -15.09 7.9557 5.1232

Cuevones Somero 1 7.4728 -9.9333 -1.7854

Cuevones Somero 2 7.4728 -9.9333 -1.7854

Cuevones Somero 3 7.4728 -9.9333 -1.7854

Cuevones Somero 4 7.4728 -9.9333 -1.7854

Donna_Nica 1 -17.075 -3.5557 -0.54499

Donna_Nica 2 -17.075 -3.5557 -0.54499

Donna_Nica 3 -17.075 -3.5557 -0.54499

Donna_Nica 4 -17.075 -3.5557 -0.54499

Donna_Nica 5 -17.075 -3.5557 -0.54499

Donna_Nica 6 -17.075 -3.5557 -0.54499

El Faro 1 -4.3342 0.48112 1.4048

El Faro 2 -4.3342 0.48112 1.4048

El Faro 3 -4.3342 0.48112 1.4048

El Faro 4 -4.3342 0.48112 1.4048

El Faro 5 -4.3342 0.48112 1.4048

El Faro 6 -4.3342 0.48112 1.4048

El Placer 01 -21.102 1.7113 1.6236

El Placer 02 -21.102 1.7113 1.6236

El Placer 03 -21.102 1.7113 1.6236

El Placer 04 -21.102 1.7113 1.6236

El Placer 05 -21.102 1.7113 1.6236

El Placer 06 -21.102 1.7113 1.6236

HNBAR002 1 -6.6515 -1.6389 -2.4622

HNBAR002 2 -6.6515 -1.6389 -2.4622

HNBAR002 3 -6.6515 -1.6389 -2.4622

HNBAR002 4 -6.6515 -1.6389 -2.4622

HNBAR002 5 -6.6515 -1.6389 -2.4622

HNBAR002 6 -6.6515 -1.6389 -2.4622

HNCYC009 3 -0.076077 11.682 3.8678

HNCYC009 4 -0.076077 11.682 3.8678

HNCYC009 5 -0.076077 11.682 3.8678

HNCYC009 6 -0.076077 11.682 3.8678

HNGUA008 1 0.77957 -1.71 2.6884

HNGUA008 2 0.77957 -1.71 2.6884

HNGUA008 3 0.77957 -1.71 2.6884

HNGUA008 4 0.77957 -1.71 2.6884

HNGUA008 5 0.77957 -1.71 2.6884

HNGUA009 1 2.2931 -1.7915 3.06

HNGUA009 3 2.2931 -1.7915 3.06

HNGUA009 4 2.2931 -1.7915 3.06

HNGUA009 5 2.2931 -1.7915 3.06

HNGUA009 6 2.2931 -1.7915 3.06

HNGUA011 1 -1.4857 -1.7908 2.3531

HNGUA011 2 -1.4857 -1.7908 2.3531

HNGUA011 3 -1.4857 -1.7908 2.3531

HNGUA011 4 -1.4857 -1.7908 2.3531

HNGUA011 5 -1.4857 -1.7908 2.3531

HNGUA011 6 -1.4857 -1.7908 2.3531

HNGUA013 1 -9.662 -1.8665 -2.7767

HNGUA013 2 -9.662 -1.8665 -2.7767

HNGUA013 3 -9.662 -1.8665 -2.7767

HNGUA013 4 -9.662 -1.8665 -2.7767

HNGUA013 5 -9.662 -1.8665 -2.7767

HNGUA013 6 -9.662 -1.8665 -2.7767

HNGUA013 7 -9.662 -1.8665 -2.7767

HNGUA014 1 8.9256 -0.39346 2.7759

HNGUA014 2 8.9256 -0.39346 2.7759

HNGUA014 3 8.9256 -0.39346 2.7759

HNGUA014 4 8.9256 -0.39346 2.7759

HNGUA014 5 8.9256 -0.39346 2.7759

HNGUA014 6 8.9256 -0.39346 2.7759

HNGUA014 7 8.9256 -0.39346 2.7759

HNGUA015 1 10.845 0.29582 2.3834

HNGUA015 2 10.845 0.29582 2.3834

HNGUA015 3 10.845 0.29582 2.3834

HNGUA015 4 10.845 0.29582 2.3834

HNGUA015 5 10.845 0.29582 2.3834

HNGUA015 6 10.845 0.29582 2.3834

HNGUA015 7 10.845 0.29582 2.3834

HNROA001X 1 2.7967 5.0982 -2.295

HNROA001X 2 2.7967 5.0982 -2.295

HNROA001X 3 2.7967 5.0982 -2.295

HNROA001X 4 2.7967 5.0982 -2.295

HNROA001X 5 2.7967 5.0982 -2.295

HNROA001X 6 2.7967 5.0982 -2.295

HNROA001X 7 2.7967 5.0982 -2.295

HNROA001X 8 2.7967 5.0982 -2.295

HNROA003 1 -2.1902 0.53849 -2.2255

HNROA003 2 -2.1902 0.53849 -2.2255

HNROA003 3 -2.1902 0.53849 -2.2255

HNROA003 4 -2.1902 0.53849 -2.2255

HNROA003 5 -2.1902 0.53849 -2.2255

HNROA003 6 -2.1902 0.53849 -2.2255

HNROA003 7 -2.1902 0.53849 -2.2255

HNROA004 1 0.98861 0.63507 -1.7366

HNROA004 2 0.98861 0.63507 -1.7366

HNROA004 3 0.98861 0.63507 -1.7366

HNROA004 4 0.98861 0.63507 -1.7366

HNROA004 5 0.98861 0.63507 -1.7366

HNROA004 6 0.98861 0.63507 -1.7366

HNROA004X 1 -4.5143 0.8067 -1.0444

HNROA004X 2 -4.5143 0.8067 -1.0444

HNROA004X 3 -4.5143 0.8067 -1.0444

HNROA004X 4 -4.5143 0.8067 -1.0444

HNROA004X 5 -4.5143 0.8067 -1.0444

HNROA004X 6 -4.5143 0.8067 -1.0444

HNROA005 1 -2.5547 -0.3984 -1.2724

HNROA005 2 -2.5547 -0.3984 -1.2724

HNROA005 3 -2.5547 -0.3984 -1.2724

HNROA005 4 -2.5547 -0.3984 -1.2724

HNROA005 5 -2.5547 -0.3984 -1.2724

HNROA005 6 -2.5547 -0.3984 -1.2724

HNROA005X 1 -4.1688 0.72507 -0.89084

HNROA005X 2 -4.1688 0.72507 -0.89084

HNROA005X 3 -4.1688 0.72507 -0.89084

HNROA005X 4 -4.1688 0.72507 -0.89084

HNROA005X 5 -4.1688 0.72507 -0.89084

HNROA005X 6 -4.1688 0.72507 -0.89084

HNROA006X 1 1.3718 1.166 -0.33588

HNROA006X 2 1.3718 1.166 -0.33588

HNROA006X 3 1.3718 1.166 -0.33588

HNROA006X 4 1.3718 1.166 -0.33588

HNROA006X 5 1.3718 1.166 -0.33588

HNROA006X 6 1.3718 1.166 -0.33588

HNROA009 1 5.923 -2.9312 1.2968

HNROA009 2 5.923 -2.9312 1.2968

HNROA009 3 5.923 -2.9312 1.2968

HNROA009 6 5.923 -2.9312 1.2968

HNROA012 1 -4.5316 -2.3281 0.46147

HNROA012 2 -4.5316 -2.3281 0.46147

HNROA012 3 -4.5316 -2.3281 0.46147

HNROA012 4 -4.5316 -2.3281 0.46147

HNROA012 5 -4.5316 -2.3281 0.46147

HNROA012 6 -4.5316 -2.3281 0.46147

HNROA013 1 2.2749 2.231 -5.012

HNROA013 2 2.2749 2.231 -5.012

HNROA013 3 2.2749 2.231 -5.012

HNROA013 4 2.2749 2.231 -5.012

HNROA013 5 2.2749 2.231 -5.012

HNROA013 6 2.2749 2.231 -5.012

HNROA014 1 1.1369 -0.28525 -2.4819

HNROA014 2 1.1369 -0.28525 -2.4819

HNROA014 3 1.1369 -0.28525 -2.4819

HNROA014 4 1.1369 -0.28525 -2.4819

HNROA014 5 1.1369 -0.28525 -2.4819

HNROA014 6 1.1369 -0.28525 -2.4819

HNROA014 7 1.1369 -0.28525 -2.4819

HNROA014 8 1.1369 -0.28525 -2.4819

HNUTI003 1 -4.2724 10.597 4.266

HNUTI003 2 -4.2724 10.597 4.266

HNUTI003 3 -4.2724 10.597 4.266

HNUTI003 4 -4.2724 10.597 4.266

HNUTI003 5 -4.2724 10.597 4.266

HNUTI003 6 -4.2724 10.597 4.266

HNUTI003 7 -4.2724 10.597 4.266

HNUTI003 8 -4.2724 10.597 4.266

HNUTI005 1 5.4462 11.927 4.633

HNUTI005 10 5.4462 11.927 4.633

HNUTI005 2 5.4462 11.927 4.633

HNUTI005 3 5.4462 11.927 4.633

HNUTI005 4 5.4462 11.927 4.633

HNUTI005 5 5.4462 11.927 4.633

HNUTI005 6 5.4462 11.927 4.633

HNUTI005 7 5.4462 11.927 4.633

HNUTI005 8 5.4462 11.927 4.633

HNUTI005 9 5.4462 11.927 4.633

HNUTI007 1 -4.2662 8.8189 2.7677

HNUTI007 2 -4.2662 8.8189 2.7677

HNUTI007 3 -4.2662 8.8189 2.7677

HNUTI007 4 -4.2662 8.8189 2.7677

HNUTI007 5 -4.2662 8.8189 2.7677

HNUTI007 6 -4.2662 8.8189 2.7677

HNUTI007 7 -4.2662 8.8189 2.7677

HNUTI007 8 -4.2662 8.8189 2.7677

Hotel Arenas 1 -4.5307 0.0093986 1.8823

Hotel Arenas 2 -4.5307 0.0093986 1.8823

Hotel Arenas 3 -4.5307 0.0093986 1.8823

Hotel Arenas 4 -4.5307 0.0093986 1.8823

Hotel Arenas 6 -4.5307 0.0093986 1.8823

Ixlache_1 24.896 -7.3617 1.0101

Ixlache_2 24.896 -7.3617 1.0101

Ixlache_3 24.896 -7.3617 1.0101

Ixlache_4 24.896 -7.3617 1.0101

Ixlache_5 24.896 -7.3617 1.0101

Ixlache_6 24.896 -7.3617 1.0101

Jardines 1 14.342 -9.4383 -1.041

Jardines 2 14.342 -9.4383 -1.041

Jardines 3 14.342 -9.4383 -1.041

Jardines 4 14.342 -9.4383 -1.041

Jardines 5 14.342 -9.4383 -1.041

La Bocana 1 7.8855 -9.5454 -2.1311

La Bocana 2 7.8855 -9.5454 -2.1311

La Bocana 3 7.8855 -9.5454 -2.1311

La Bocana 4 7.8855 -9.5454 -2.1311

La Bocana 5 7.8855 -9.5454 -2.1311

La Catedral 5m 1 2.7661 -10.226 -2.3464

La Catedral 5m 2 2.7661 -10.226 -2.3464

La Catedral 5m 3 2.7661 -10.226 -2.3464

La Catedral 5m 4 2.7661 -10.226 -2.3464

La Catedral 5m 5 2.7661 -10.226 -2.3464

La Catedral 5m 6 2.7661 -10.226 -2.3464

La Catedral Posterior 1 17.03 -8.9361 -1.086

La Catedral Posterior 2 17.03 -8.9361 -1.086

La Catedral Posterior 3 17.03 -8.9361 -1.086

La Catedral Posterior 4 17.03 -8.9361 -1.086

La Catedral Posterior 5 17.03 -8.9361 -1.086

La Catedral Posterior 6 17.03 -8.9361 -1.086

Limones 1 11.177 -9.3104 -1.772

Limones 2 11.177 -9.3104 -1.772

Limones 3 11.177 -9.3104 -1.772

Limones 4 11.177 -9.3104 -1.772

Limones 5 11.177 -9.3104 -1.772

Mahahual 1 -13.788 -0.12965 0.30356

Mahahual 2 -13.788 -0.12965 0.30356

Mahahual 3 -13.788 -0.12965 0.30356

Mahahual 4 -13.788 -0.12965 0.30356

Mahahual 5 -13.788 -0.12965 0.30356

Mahahual 6 -13.788 -0.12965 0.30356

Mahahual Centro 1 -0.12649 0.78392 1.8612

Mahahual Centro 2 -0.12649 0.78392 1.8612

Mahahual Centro 3 -0.12649 0.78392 1.8612

Mahahual Centro 4 -0.12649 0.78392 1.8612

Mahahual Centro 5 -0.12649 0.78392 1.8612

Mahahual Centro 6 -0.12649 0.78392 1.8612

Mahahual Cresta 1 23.916 2.3917 4.6025

Mahahual Cresta 2 23.916 2.3917 4.6025

Mahahual Cresta 3 23.916 2.3917 4.6025

Mahahual Cresta 4 23.916 2.3917 4.6025

Mahahual Cresta 5 23.916 2.3917 4.6025

Mahahual Cresta 6 23.916 2.3917 4.6025

Mar F2 1 2.9468 -7.8235 -2.589

Mar F2 2 2.9468 -7.8235 -2.589

Mar F2 3 2.9468 -7.8235 -2.589

Mar F2 4 2.9468 -7.8235 -2.589

Mar F2 5 2.9468 -7.8235 -2.589

Mar F2 6 2.9468 -7.8235 -2.589

Mar F4 1 -9.401 -8.6493 -3.9969

Mar F4 2 -9.401 -8.6493 -3.9969

Mar F4 3 -9.401 -8.6493 -3.9969

Mar F4 4 -9.401 -8.6493 -3.9969

Mar F4 5 -9.401 -8.6493 -3.9969

Mar F4 6 -9.401 -8.6493 -3.9969

Mar F5 1 2.9468 -7.8235 -2.589

Mar F5 2 2.9468 -7.8235 -2.589

Mar F5 3 2.9468 -7.8235 -2.589

Mar F5 4 2.9468 -7.8235 -2.589

Mar F5 5 2.9468 -7.8235 -2.589

Mar F5 6 2.9468 -7.8235 -2.589

Mar P1 1 25.327 -6.3269 -0.037247

Mar P1 2 25.327 -6.3269 -0.037247

Mar P1 3 25.327 -6.3269 -0.037247

Mar P1 4 25.327 -6.3269 -0.037247

Mar P1 5 25.327 -6.3269 -0.037247

Mar P1 6 25.327 -6.3269 -0.037247

Microatolones 5m 1 2.2478 -7.3366 -0.67386

Microatolones 5m 2 2.2478 -7.3366 -0.67386

Microatolones 5m 3 2.2478 -7.3366 -0.67386

Microatolones 5m 4 2.2478 -7.3366 -0.67386

Microatolones 5m 5 2.2478 -7.3366 -0.67386

Microatolones 5m 6 2.2478 -7.3366 -0.67386

Mosquitero 01 -18.452 3.9488 2.3924

Mosquitero 02 -18.452 3.9488 2.3924

Mosquitero 03 -18.452 3.9488 2.3924

Mosquitero 04 -18.452 3.9488 2.3924

Mosquitero 05 -18.452 3.9488 2.3924

Mosquitero 06 -18.452 3.9488 2.3924

MS 01 -19.352 -0.98172 0.19239

MS 02 -19.352 -0.98172 0.19239

MS 03 -19.352 -0.98172 0.19239

MS 04 -19.352 -0.98172 0.19239

MS 05 -19.352 -0.98172 0.19239

MS 06 -19.352 -0.98172 0.19239

MX1028 01 -19.626 5.8223 3.1631

MX1028 02 -19.626 5.8223 3.1631

MX1028 03 -19.626 5.8223 3.1631

MX1028 04 -19.626 5.8223 3.1631

MX1028 05 -19.626 5.8223 3.1631

MX1028 06 -19.626 5.8223 3.1631

MX1037 01 -16.086 2.4773 1.7261

MX1037 02 -16.086 2.4773 1.7261

MX1037 03 -16.086 2.4773 1.7261

MX1037 04 -16.086 2.4773 1.7261

MX1037 05 -16.086 2.4773 1.7261

MX1037 06 -16.086 2.4773 1.7261

MX1042 1 -9.686 0.6071 0.26718

MX1042 2 -9.686 0.6071 0.26718

MX1042 3 -9.686 0.6071 0.26718

MX1042 4 -9.686 0.6071 0.26718

MX1042 5 -9.686 0.6071 0.26718

MX1042 6 -9.686 0.6071 0.26718

MX1043 1 -8.3163 2.2387 -1.2552

MX1043 2 -8.3163 2.2387 -1.2552

MX1043 3 -8.3163 2.2387 -1.2552

MX1043 4 -8.3163 2.2387 -1.2552

MX1043 5 -8.3163 2.2387 -1.2552

MX1043 6 -8.3163 2.2387 -1.2552

MX1065 01 -16.884 -2.3248 0.056977

MX1065 02 -16.884 -2.3248 0.056977

MX1065 03 -16.884 -2.3248 0.056977

MX1065 04 -16.884 -2.3248 0.056977

MX1065 05 -16.884 -2.3248 0.056977

MX1065 06 -16.884 -2.3248 0.056977

MX1065 1 -16.884 -2.3248 0.056977

MX1065 2 -16.884 -2.3248 0.056977

MX1065 3 -16.884 -2.3248 0.056977

MX1065 4 -16.884 -2.3248 0.056977

MX1065 5 -16.884 -2.3248 0.056977

MX1065 6 -16.884 -2.3248 0.056977

MX1132 1 17.658 -8.877 -1.033

MX1132 2 17.658 -8.877 -1.033

MX1132 3 17.658 -8.877 -1.033

MX1132 4 17.658 -8.877 -1.033

MX1132 5 17.658 -8.877 -1.033

MX1132 6 17.658 -8.877 -1.033

MX1136 1 15.685 -0.13926 3.7623

MX1136 2 15.685 -0.13926 3.7623

MX1136 3 15.685 -0.13926 3.7623

MX1136 4 15.685 -0.13926 3.7623

MX1136 5 15.685 -0.13926 3.7623

MX1136 6 15.685 -0.13926 3.7623

MX2067 1 -12.247 -1.7677 0.31649

MX2067 2 -12.247 -1.7677 0.31649

MX2067 3 -12.247 -1.7677 0.31649

MX2067 4 -12.247 -1.7677 0.31649

MX2067 5 -12.247 -1.7677 0.31649

MX2067 6 -12.247 -1.7677 0.31649

MXXCK01 1 -11.249 -4.4422 -0.26574

MXXCK01 2 -11.249 -4.4422 -0.26574

MXXCK01 3 -11.249 -4.4422 -0.26574

MXXCK01 4 -11.249 -4.4422 -0.26574

MXXCK01 5 -11.249 -4.4422 -0.26574

MXXCK01 6 -11.249 -4.4422 -0.26574

MXXCK02 1 -5.7516 -3.9256 0.19856

MXXCK02 2 -5.7516 -3.9256 0.19856

MXXCK02 3 -5.7516 -3.9256 0.19856

MXXCK02 4 -5.7516 -3.9256 0.19856

MXXCK02 5 -5.7516 -3.9256 0.19856

MXXCK02 6 -5.7516 -3.9256 0.19856

Nizuc F3 1 -11.198 -10.794 -4.3368

Nizuc F3 2 -11.198 -10.794 -4.3368

Nizuc F3 3 -11.198 -10.794 -4.3368

Nizuc F3 4 -11.198 -10.794 -4.3368

Nizuc F3 5 -11.198 -10.794 -4.3368

Nizuc F3 6 -11.198 -10.794 -4.3368

P. Paila 1 -9.6546 3.2035 2.1367

P. Paila 2 -9.6546 3.2035 2.1367

P. Paila 3 -9.6546 3.2035 2.1367

P. Paila 4 -9.6546 3.2035 2.1367

P. Paila 5 -9.6546 3.2035 2.1367

P. Paila 6 -9.6546 3.2035 2.1367

PM-F2 1 -9.5841 -11.057 -3.7484

PM-F2 2 -9.5841 -11.057 -3.7484

PM-F2 3 -9.5841 -11.057 -3.7484

PM-F2 4 -9.5841 -11.057 -3.7484

PM-F2 5 -9.5841 -11.057 -3.7484

PM-F2 6 -9.5841 -11.057 -3.7484

Puerto Angel 1 -9.0327 -1.9214 1.0848

Puerto Angel 2 -9.0327 -1.9214 1.0848

Puerto Angel 3 -9.0327 -1.9214 1.0848

Puerto Angel 4 -9.0327 -1.9214 1.0848

Puerto Angel 5 -9.0327 -1.9214 1.0848

Puerto Angel 6 -9.0327 -1.9214 1.0848

Punta Allen Centro 1 -13.075 2.7192 2.0253

Punta Allen Centro 2 -13.075 2.7192 2.0253

Punta Allen Centro 3 -13.075 2.7192 2.0253

Punta Allen Centro 4 -13.075 2.7192 2.0253

Punta Allen Centro 5 -13.075 2.7192 2.0253

Punta Allen Centro 6 -13.075 2.7192 2.0253

Punta Allen Norte 1 -11.336 2.7575 2.3087

Punta Allen Norte 2 -11.336 2.7575 2.3087

Punta Allen Norte 3 -11.336 2.7575 2.3087

Punta Allen Norte 4 -11.336 2.7575 2.3087

Punta Allen Norte 5 -11.336 2.7575 2.3087

Punta Allen Norte 6 -11.336 2.7575 2.3087

Tampalam Centro 01 -8.9728 6.3376 4.5926

Tampalam Centro 02 -8.9728 6.3376 4.5926

Tampalam Centro 03 -8.9728 6.3376 4.5926

Tampalam Centro 04 -8.9728 6.3376 4.5926

Tampalam Centro 05 -8.9728 6.3376 4.5926

Tampalam Centro 06 -8.9728 6.3376 4.5926

Tanchacte Sur 1 12.878 -9.5816 -1.1585

Tanchacte Sur 2 12.878 -9.5816 -1.1585

Tanchacte Sur 3 12.878 -9.5816 -1.1585

Tanchacte Sur 4 12.878 -9.5816 -1.1585

Tanchacte Sur 5 12.878 -9.5816 -1.1585

XM04 01 -28.007 1.4612 0.60559

XM04 02 -28.007 1.4612 0.60559

XM04 03 -28.007 1.4612 0.60559

XM04 04 -28.007 1.4612 0.60559

XM04 05 -28.007 1.4612 0.60559

XM04 06 -28.007 1.4612 0.60559

XYSF1 01 -11.667 -2.8749 -0.27614

XYSF1 02 -11.667 -2.8749 -0.27614

XYSF1 03 -11.667 -2.8749 -0.27614

XYSF1 04 -11.667 -2.8749 -0.27614

XYSF1 05 -11.667 -2.8749 -0.27614

XYSF1 06 -11.667 -2.8749 -0.27614

*Relationships between dbRDA coordinate axes and orthonormal X variables*

*(multiple partial correlations)*

Variable dbRDA1 dbRDA2 dbRDA3

Depth -0.969 -0.052 -0.243

Wave_exposure -0.173 -0.562 0.809

Hurricane_impact 0.178 -0.826 -0.535

*Weights*

*(Coefficients for linear combinations of X's in the formation of dbRDA coordinates)*

Variable dbRDA1 dbRDA2 dbRDA3

Depth -12.51 -0.83655 -1.4264

Wave_exposure -1.2782 -3.0693 3.1067

Hurricane_impact 0.68559 -5.2524 -2.3553

*Outputs*

Plot: Graph67
